# Preoperative and perioperative factors that predict graft failure 1 year after Descemet membrane endothelial keratoplasty

**DOI:** 10.1371/journal.pone.0352687

**Published:** 2026-07-24

**Authors:** Lucas Mastrangelo, Jules Leterrier, Christophe Goetz, Yinka Zevering, Jean-Marc Perone

**Affiliations:** 1 Ophthalmology Department, Regional Hospital Center of Metz-Thionville, Mercy Hospital, Metz, Grand-Est, France; 2 Clinical Research Support Unit, Regional Hospital Center of Metz-Thionville, Mercy Hospital, Metz, Grand-Est, France; University of Missouri-Columbia, UNITED STATES OF AMERICA

## Abstract

**Purpose:**

To identify pre/perioperative factors that predict graft failure after Descemet membrane endothelial keratoplasty (DMEK).

**Methods:**

This retrospective cohort study included consecutive eyes that underwent DMEK in 2015–2023 in a regional referral hospital and were followed for at least 12 months. DMEK-graft failure was defined as need for regrafting during follow-up. Univariable analysis of graft-failure associations with 20 covariates was performed. Hierarchical multivariable analysis was conducted for DMEK-graft failure with covariates whose univariable-analysis p-values were ≤0.15. Posthoc univariable analyses were performed to elucidate the mechanisms by which covariates promoted DMEK-graft failure.

**Results:**

171 eyes (129 patients) with mostly Fuchs endothelial corneal dystrophy (94%) were included. Median (range) follow-up was 24 (12–29) months, during which 15 grafts (9%) failed. On univariable analyses, graft failure associated with eight variables, including long (≥25 mm) preoperative axial length (AXL): 40% of graft-failure eyes had AXL ≥ 25 mm vs. 13% for graft-success eyes (p = 0.01). On multivariable analysis, graft failure was predicted by AXL ≥ 25 mm (OR=5.70; 95%CI = 1.27–25.68; p = 0.02), younger graft-donor age (OR=0.93; 95%CI = 0.88–0.99; p = 0.02), graft-unscrolling/positioning difficulties (OR=6.93; 95%CI = 1.58–30.48; p = 0.01), and major graft detachment (OR=6.28; 95%CI = 1.36–28.96; p = 0.02). The model accounted for 14% of total graft-failure variance. Posthoc univariable analysis showed that eyes with AXL ≥ 25 experienced graft-unscrolling/positioning difficulties more often than shorter eyes (27% vs. 10%, p = 0.0497).

**Conclusions:**

Eyes with long AXL (≥25 mm) may be more prone to DMEK-graft failure than shorter eyes. This may reflect the deep anterior chamber, which can complicate graft unscrolling and/or graft positioning. The additional graft handling may induce endothelial-cell loss and subsequent graft failure. Younger donor age weakly predicted DMEK-graft failure, possibly due to tighter scrolling of younger grafts. Major graft detachment strongly predicted DMEK-graft failure. This could reflect surgical and/or rebubbling-induced endothelial damage and/or pre-existing graft weakness.

## Introduction

Descemet membrane endothelial keratoplasty (DMEK) is a corneal-transplantation method that involves peeling off a disc of diseased corneal endothelium and its overlying Descemet membrane, replacing it with a disc of donor tissue composed of healthy endothelium and Descemet membrane, and pinning the graft against the recipient cornea with a bubble of air or 20% sulfur hexafluoride (SF6) that wanes over the next few days and weeks, respectively [1,2]. DMEK is the gold standard for corneal-endothelial pathologies [3], including Fuchs endothelial corneal dystrophy (FECD), a genetic condition characterized by progressive loss of endothelial cells and eventual corneal stromal swelling that induces halos, discomfort, and visual-acuity loss [4]. DMEK is also used to treat bullous keratopathy (BK), where ocular surgery such as phacoemulsification has traumatized the endothelium [3], and graft failure after previous keratoplasties, including Descemet stripping automated endothelial keratoplasty (DSAEK) and penetrating keratoplasty (PKP) [5].

The thinness of the DMEK graft (15 µm) results in superior visual outcomes compared to DSAEK (45–180 µm-thick grafts) and PKP (full-thickness grafts) [6–9]. However, this thinness together with the elasticity of Descemet membrane also means DMEK grafts form tight scrolls that must be unfurled in the recipient anterior chamber. A common approach is to gently tap the cornea with two 27-gauge cannulas, after which the unfurled graft is positioned against the exposed recipient stroma with the air or gas bubble. However, this procedure can be complicated by incomplete graft unscrolling, graft rescrolling, and difficult graft positioning, which require additional maneuvers that further stress the graft and exacerbate donor endothelial-cell loss (ECL) [10–13]. The scroll-prone graft also tends to detach from the recipient bed, which can require early postoperative rebubbling. However, rebubbling could potentially also induce ECL [11,14–21]. Since (i) DMEK associates with 30–50% graft ECL in the first 6 postoperative months [22,23], (ii) DMEK accelerates aging-related ECL from 0.6–0.7%/year to ~4%/year [22,23], and (iii) postoperative graft endothelial-cell counts of 250–500 cells/mm^2^ associate strongly with graft failure [22–24], surgeons remain concerned about DMEK-graft longevity. Indeed, up to 13% and 6% of DMEK grafts respectively fail in the first few postoperative months and within the next 5 years [25].

Given these concerns, it would be useful to know which preoperative and perioperative factors predict DMEK-graft failure, since this could help identify at-risk patients and/or strategies that reduce DMEK-graft failure. However, few studies have focused on DMEK-graft failure specifically (S1 Table) [20,26–37]. Most studies instead use ECL, graft detachment, or rebubbling as surrogate measures of graft failure (e.g., [38–43]), probably because graft-failure sample sizes are generally small. However, ECL is generally unmeasurable in early-failure cases and only ~30% of graft detachment/rebubbling cases actually undergo graft failure [20]. Moreover, graft detachment and surgery-induced endothelial decompensation cannot explain all DMEK-graft failures [44,45]: multiple studies show that graft failure can also be caused by undetected ultrastructural graft aberrations and graft disease (e.g., early-stage pseudoexfoliation-associated keratopathy and corneal guttata) [45–49], disease recurrence [50], immune rejection, intraocular-pressure elevation, and wound dehiscence [25,50]. Thus, the surrogate variables do not fully represent DMEK-graft failure. Moreover, some of the few studies on DMEK-graft failure itself conducted univariable rather than multivariable analyses (S1 Table) [20,26–37]. Some potential risk factors have also been poorly researched, including ocular anatomical variables such as axial length (AXL) or anterior-chamber depth (ACD): while two studies suggest that they could affect DMEK-graft unfolding and promote ECL [51,52], others have not observed this [53–55] and their contribution to DMEK-graft failure has not been studied. Similarly, although 20% SF6 is increasingly being used instead of air alone for endotamponade due to longer bubble persistence [56], the effect of using SF6 on DMEK-graft failure has rarely been assessed [27,28]. Moreover, although DMEK graft-unscrolling/positioning difficulties can induce ECL [10–13], their direct influence on graft failure has not been examined.

To identify factors that could specifically shape DMEK-graft failure, we subjected our unselected cohort of DMEK patients to univariable and multivariable analyses to determine the relationships between DMEK-graft failure and 20 pre/perioperative factors, including ocular-anatomy variables, SF6 use, and graft-unscrolling/positioning difficulties. Posthoc analyses were also conducted to elucidate potential underlying mechanisms by which the predictive pre/perioperative factors shape DMEK-graft failure.

## Methods

### Study design and ethics

This retrospective single-center cohort study was conducted in the Ophthalmology Department of the Metz-Thionville Regional Hospital (Grand Est, France). It was approved by the Ethics Committee of the French Society of Ophthalmology (Institutional Review Board No. 00008855) and registered on ClinicalTrials.gov (Identifier: NCT06859411). All procedures were conducted in accordance with the principles of the Declaration of Helsinki. Before surgery, all patients were informed that their surgery-related data might be used for research. All consented to this possibility in writing. The consent procedure was conducted according to the reference methodology MR-004 of the National Commission for Information Technology and Liberties of France (No. 588909 v1). The data were accessed for research purposes on 7 April 2025. Only the first and corresponding authors had access to information that could identify individuals during or after data collection.

### Patient selection

The prospectively maintained medical records were searched for all consecutive adult (≥18 years) patients who underwent primary DMEK in 1 October 2015–30 April 2023 and were followed for at least 12 months. Patients whose surgeries involved graft-dissection problems (tearing or splitting of the graft, or dissection resulting in an imperfect graft shape) were excluded because graft-preparation difficulties can damage the graft and increase graft failure [57] and we wanted to focus on the outcomes of apparently viable grafts. This was because many centers use eye bank-prepared DMEK grafts [38] and we wanted our study to be relevant to this setting as well. Patients with history of vitrectomy were excluded because vitrectomy also associates with more graft failure [58]. One eye that developed unusually severe endothelial damage was excluded because it was not clear whether the graft failure was due to rejection or viral infection or both. Other rejection cases were not *a priori* excluded. Eyes with missing AXL and/or ACD data were also excluded. To assess the effect of these exclusions on our findings, we repeated our multivariable analysis with all consecutive patients.

### Preoperative, perioperative, and postoperative measurements

Preoperative AXL, ACD, and lens thickness of the operative eye were measured with a biometer (IOLMaster 700; Zeiss Co., Oberkochen, Germany). BSCVA (expressed in logMAR) was measured before and 8 and 15 days and 1, 3, 6, and 12 months after surgery. Anterior-segment optical coherence tomography (NIDEK with a special module; Nidek Co., Gamagori Aichi, Japan) was conducted at all postoperative timepoints to determine graft adherence. Operative time from starting graft dissection to placing the suture at the end of surgery was measured by a nurse with a stopwatch. Preoperative graft ECD was determined by manual counting under an optic microscope by an eye-bank technician followed by a recount by another technician.

### Surgical techniques, postoperative care, and follow-up

During the preoperative consultation, all patients underwent lower peripheral iridotomy with Nd:YAG Laser (Laser ex-Super Q; Ellex Europe, Medical Quantel, Cournon d’Auvergne, France) at the 6 o’clock position to prevent pupillary block during and after DMEK surgery. All surgeries were performed by the same experienced surgeon (JMP). General anesthesia was used unless it was contraindicated, in which case surgery was performed under peribulbar block with a 50:50 mixture of ropivacaine 7.5 mg/mL and lidocaine 2% that was administered with a 23-gauge Atkinson cannula.

DMEK was conducted as described previously [2]. Unprepared corneoscleral buttons were obtained from the tissue banks in Besançon or Nancy, France. All were preserved in CorneaMax organ-culture medium (Eurobio; Courtaboeuf, France) at 31°C. All had a requested endothelial-cell density (ECD) of >2200 cells/mm². These preoperative ECD measurements were made just before transport, which occurred 3–4 weeks after button harvest. For transport, the buttons were placed in dextran-containing transport medium (CorneaJet; Eurobio) and shipped to our hospital the day before surgery. They were exposed to the transport medium for 3 days. DMEK grafts were generated from the corneoscleral buttons immediately before surgery. Specifically, in the operating room, each graft was trephined to an 8 mm-diameter disc with Hanna’s microtrephine (Busin Punch 17200D 8-mm single use; Moria SA, Antony, France). The Descemet membrane-endothelium complex was then manually stripped from the corneal stroma with disposable curved forceps (Single Use Tying Forceps Curved 5 mm Platform 17501; Moria SA) under a microscope. The graft was stained with Trypan Blue (Vision Blue, 0.5-mL syringe; DORC [Dutch Ophthalmic Research Center], Zuidland, Netherlands]), marked with “F” or “F*” on its stromal side with a gentian-violet surgical pen (Devon Skin Marker; Covidien, Mansfield, USA), and placed into a customized injector (30G Curved cannula for air injection; DORC).

If the recipient eye had concomitant anterior corneal surface pathology, superficial keratectomy was conducted with alcohol delamination, followed by polishing with a cellulose sponge and irrigation. The central surface of the recipient cornea was marked with an 8 mm-diameter circular marker and the main paracentesis was placed supero-temporally (right eye) or supero-nasally (left eye) with a 2.2-mm blade (Securityblade BD, Xstar 2.2-mm, 45 degrees, 37822; Beaver-Visitec International, Waltham, USA). A second incision was made with a Worst 15 blade (Ophthalmic Knife 15 degrees; ALCON, Rueil Malmaison, France). A 9 mm-diameter central descemetorhexis was performed along the circular marks on the cornea with an inverted Sinskey Price hook (Single Use Price Reverse Hook Sim 17302; Moria SA) and an inverted spatula (90th single use Spatula 17303; Moria SA) under sterile air infusion. The main incision was enlarged to 4 mm and the graft was injected into the anterior chamber via the DORC injector. The graft was unscrolled by applying external corneal pressure and taps with two 27-gauge Rycroft cannulas. Once positioned, a sterile air or 20% SF6 bubble was injected into the anterior chamber to secure the graft. We used air for endotamponade until September 2020, at which point we switched to SF6 for all new eyes. The main incision was sutured with one point of Nylon 10.0, which was subsequently buried.

All phakic eyes also underwent simultaneous cataract surgery (triple-DMEK). All remaining eyes were pseudophakic before DMEK. In triple-DMEK cases, phacoemulsification was performed immediately before DMEK using a standard supracapsular “garde à vous” technique [59] with the Stellaris PC (Bausch and Lomb, Aliso Viejo, CA, USA). A Zeiss CT Asphina 409MV intraocular lens was implanted in the capsular bag, targeting a residual myopia of 0.5–1.0 diopters to counteract the expected post-DMEK hyperopic shift [60]. Carbachol 0.01% (Miostat; ALCON) was used to induce miosis, which facilitated the subsequent DMEK procedure.

After DMEK, 1 ml cefuroxime 50 mg (Aprokam; Thea Laboratory, Clermont-Ferrand, France) was injected intracamerally into the air/gas bubble and acetazolamide 500 mg (Diamox; Sanofi, Gentilly, France) was infused. The patients were treated four times/day with eyedrops containing dexamethasone, neomycin, and polymyxine B (Maxidrol; ALCON), the non-steroidal anti-inflammatory drug indometacine 0.1% (Indocollyre; Bausch and Lombe, Montpellier, France), and a vitamin A-containing ophthalmic ointment (Vitamin A dulcis; Laboratoire Allergan, Courbevoie, France) for 1 month. Indometacine and the ointment were then stopped and Maxidrol was replaced with low-dose corticosteroid eye drops (Flucon; Novartis Pharma, Rueil Malmaison, France), which were administered three times/day for 2 months, twice/day for the next year, and then once/day for generally 5 years.

After surgery, the patients were followed up at days 8 and 15, months 1, 3, 6, and 12, and every year thereafter. Patients who developed CME were treated with oral acetazolamide (Diamox; one 250 mg tablet three times/day for 1 month) and indometacine (four times/day for 1 month) or, in complex cases, with intravitreal injection of dexamethasone (Ozurdex 700 µg implant i-vitr; Laboratoire Allergan). Patients with graft rejection were treated with dexamethasone with oxytetracycline ointment (Sterdex; Thea, Clermont-Ferrand, France; twice/day) and Maxidrol eyedrops (Alcon; 12 times/day for 1 week followed by tapering for several weeks).

If ≥30% of the graft had detached and/or the detachment threatened the visual axis, it was considered major graft detachment. In all cases, as well as in some early cases where the detachment was more minor, rebubbling with sterile air was conducted under topical anesthesia. Rebubbling was repeated if considered necessary.

If non-adhesion continued despite up to four rebubblings, corneal edema persisted after 3 months, and/or new corneal edema arose during follow-up, the graft was considered to have failed and regrafting with DMEK or DSAEK was planned.

### Collected variables and definitions

The data of the following variables were collected: patient age and sex; operative eye side; indication; preoperative recipient AXL, ACD, and lens thickness (note that the latter data could not be collected for eyes that were pseudophakic before DMEK); preoperative and 12-month postoperative BSCVA; graft-donor age and preoperative ECD; use of general anesthesia; use of triple-DMEK; graft-unscrolling/positioning difficulties; use of SF6 for endotamponade; operative time; presence of a bubble under the iris; presence of major graft detachment; number of rebubblings (0, 1, ≥ 2); graft rejection; development of CME; and graft failure. Graft-unscrolling/positioning difficulties were defined according to Maier et al. [12], namely, difficult unfolding and centering (duration longer than 5 minutes) that required repeated air injection and balanced salt solution exchange or even direct manipulation of the graft with forceps. Major graft detachment was defined as detachment that affected the graft center and/or >30% of the graft surface and therefore required rebubbling. Graft failure was defined as need for a new graft during follow-up due to any reason, including failure caused by rejection. Patients whose graft failed but eschewed a regraft were still considered graft-failure cases.

### Statistical analysis

**Fig 1:**
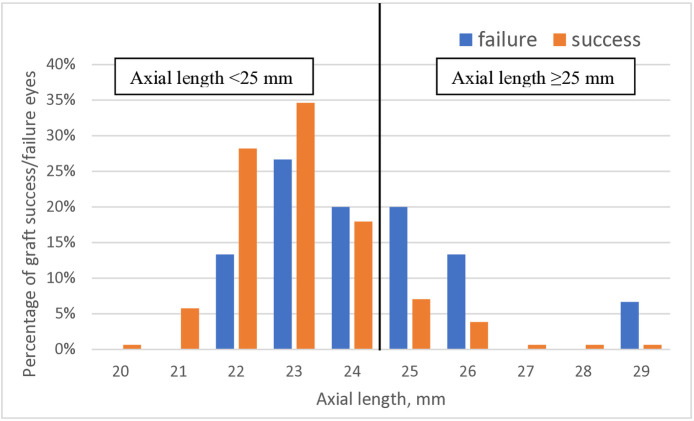
Distribution of axial length in eyes with and without graft failure (*n* = 170).

Continuous data were expressed as median and interquartile range (IQR). Categorical data were expressed as *n* (%). Since our exploratory univariable analyses showed that longer AXL associated with graft failure (see Results), and eyes with ≥25-mm AXL seemed markedly more prone to graft failure than eyes with AXL < 25 mm (Fig 1), we expressed AXL as both a continuous and categorical variable (i.e., < 25 mm and ≥25 mm). The univariable relationships between graft failure and the 20 preoperative and perioperative variables were then determined with generalized linear regression with a random factor on the patient: this test was used rather than standard univariable analyses (e.g. Fisher’s exact test) because a third of patients underwent bilateral DMEK, which contravenes the assumption of sample independence. All independent variables that had a p-value of ≤0.15 on univariable analysis were considered for inclusion in a generalized multiple linear regression analysis to determine their ability to predict graft failure. This regression analysis was also conducted with a random factor on the patient to account for the bilateral DMEK cases. Before multivariable analysis was conducted, eigenvalues and eigenvectors were determined to assess collinearity between the independent variables. If collinearity was detected between two independent variables, the most clinically relevant variable was retained in the multivariable analysis. AXL was expressed as a categorical variable in the multivariable analysis to determine the clinical applicability of the ≥ 25-mm threshold. However, this analysis was also repeated with AXL expressed as a continuous variable. All multivariable-analysis data were expressed as Odds Ratio (OR) and 95% confidence intervals (95%CI). Missing data were ignored in all analyses. To depict the time course of graft failures, Kaplan-Meier survival analysis was conducted. To assess the effect of excluding cases with graft-dissection problems, vitrectomy, missing data, and complicated rejection/infection on our multivariable-analysis findings, we repeated this analysis with all consecutive patients. P-values <0.05 were considered to indicate statistical significance. All statistical analyses were performed with SAS software (version 9.4, SAS Inst., Cary, NC, USA).

### Posthoc statistical analyses

Our multivariable analyses revealed that two variables, namely, longer AXL and graft-unscrolling/positioning difficulties, were strong predictors of graft failure (see Results). We hypothesized that longer AXL might promote graft failure indirectly by inducing graft unscrolling/positioning difficulties because (i) longer AXL correlates positively with deeper ACD [61–63] and (ii) studies suggest that DMEK-graft unscrolling is difficult in deep anterior chambers because the graft spins on tapping rather than unfurling [14,64–66]. To test our hypothesis, the univariable relationships between all significant predictors in the multivariable analysis were determined with posthoc generalized linear regressions with a random factor on the patient. To determine whether the relationships detected by these univariable analyses could have confounded the first multivariable analyses, posthoc multivariable analyses that excluded single graft-failure predictors were also conducted.

The relationships between AXL and the collinear variables that were excluded from the first multivariable analyses due to collinearity were also determined with posthoc generalized linear regressions with a random factor on the patient.

## Results

**Fig 2:**
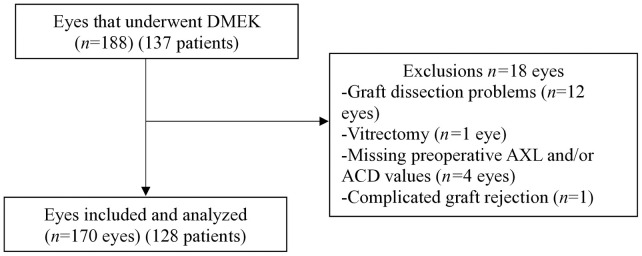
Patient inclusion flowchart. ACD, anterior chamber depth; AXL, axial length. DMEK, Descemet membrane endothelial keratoplasty.

During the study period, 188 eyes of 137 patients underwent DMEK. Of these, 17 eyes from eight patients were excluded due to graft-dissection problems (*n* = 12), a history of vitrectomy (*n* = 1), and missing preoperative AXL and/or ACD values (*n* = 4). One unusual case with extremely severe endothelial damage that required regraft with PKP was excluded because it was not possible to determine whether rejection and/or viral endothelitis was the cause of graft failure. Thus, 170 eyes of 128 patients were included in this study (Fig 2). The median (IQR; range) follow-up duration of these 170 eyes was 24 (12–24; 12–29) months.

### Preoperative and operative cohort characteristics and postoperative outcomes

The data were complete for all eyes except preoperative BSCVA was missing for one eye and preoperative lens thickness was missing for two triple-DMEK eyes (and all 82 pseudophakic-DMEK eyes). The 128 patients had a median age of 72 years, 66% were women, and 33% underwent bilateral DMEK. The indications in the 170 operative eyes were FECD (94%), pseudophakic BK (3%), regraft (3%), and post-uveitis (1%). Median preoperative AXL was 23 mm, and 144 (85%) and 26 (15%) eyes had AXL < 25 mm and AXL ≥ 25 mm, respectively (Fig 1). Median graft donor age was 74 years. Other descriptive data are shown in Table 1.

**Table 1:** Preoperative, perioperative, and postoperative characteristics of the cohort (*n* = 170).

Characteristic	Missing values	*n* (%)	Median (IQR)
Patient age, y	0		72 (66–78)
Patient female sex	0	113 (66)	
Operated eye on right	0	93 (55)	
Indication FECD PBK Regraft Post-uveitis	0	159 (94) 5 (3) 5 (3) 1 (1)	
Preop AXL, mm	0		23 (23–25)
<25mm ≥25 mm		144 (85) 26 (15)	
Preop ACD, mm	0		3.20 (2.84–3.88)
Preop LT, mm	84†		4.76 (4.57–5.03)
Preop BSCVA, logMAR	1		0.5 (0.4–0.7)
Donor age, y	0		74 (68–82)
Donor ECD, cells/mm^2^	0		2540 (2400–2680)
General anesthesia	0	166 (98)	
Superficial keratectomy	0	0	
Triple-DMEK	0	88 (52)	
Graft-unscroll/positioning difficulties	0	22 (13)	
SF6 use	0	63 (37)	
Operative time, min	0		35 (30–40)
Bubble under iris	0	3 (2)	
Major graft detachment	0	25 (15)	
Rebubbling	0	55 (32)	
No. rebubblings 0 1 ≥2	0	115 (67) 37 (22) 18 (11)	
Cystoid macular edema	0	13 (8)	
Graft rejection	0	0 (0)	
Graft failure within 12 months	0	15 (9)	

†Lens-thickness values were not obtained for the 82 pseudophakic-DMEK cases, since these eyes had an IOL rather than a natural lens at the time of DMEK. Lens-thickness values for two triple-DMEK cases were missing.

ACD, anterior chamber depth; AXL, axial length; BSCVA, best spectacle-corrected visual acuity; FECD, Fuchs endothelial corneal dystrophy; IQR, interquartile range; LT, lens thickness; PBK, pseudophakic bullous keratopathy; preop, preoperative; SF6, sulfur hexafluoride; y, years.

**Fig 3:**
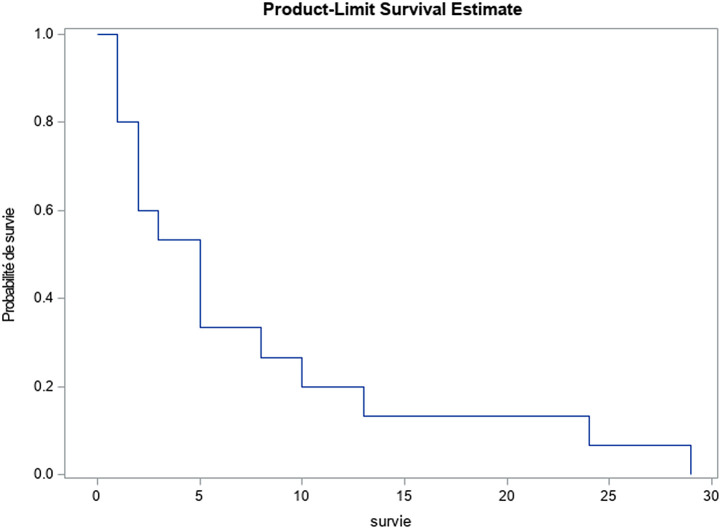
Time point at which the 15 DMEK grafts failed during follow-up. The plot was generated with Kaplan-Meier analysis.

In 22 eyes (13%), we experienced graft-unscrolling/positioning difficulties that lengthened this procedure to more than 5 minutes, necessitating repeated air injection with balanced salt solution exchange and in some cases requiring direct graft manipulation with forceps. SF6 was used for endotamponade in 37% and median operative time was 35 minutes. A bubble appeared under the iris in three eyes (2%). Major graft detachment necessitating rebubbling was observed in 25 eyes (15%). Another 30 eyes (18%) from early in the series were also rebubbled despite more minor graft detachment. Thus, 55 eyes (32%) underwent rebubbling. A single rebubble and ≥2 rebubbles were conducted in 37 (22% of all eyes) and 18 (11%) eyes, respectively. There were 13 cases of CME (8%), all of which resolved with treatment, and no cases of rejection. During follow-up, 15 (9%) of the eyes had experienced graft failure (Table 1). Of these 15 grafts, seven (47%), three (20%), two (13%), and three (20%) failed at ≤3 months, 4–6 months, 7–12 months, and >12 months, respectively. The median (IQR) duration to graft failure was 5 (2–9) months. Fig 3 shows when the 15 grafts failed during follow-up. The median 12-month BSCVA of the eyes whose graft survived was 0.08 logMAR.

### Univariable and multivariable associations between graft failure and pre/perioperative variables

Generalized linear regression analysis with a factor on the patient (which accounted for the bilateral-DMEK cases) showed that the eyes that developed graft failure had significantly longer median preoperative AXL (25 *vs.* 23 mm for the graft-success eyes, p = 0.02). The graft-failure eyes were also 3-fold more likely to have a preoperative AXL of ≥25 mm than the graft-success eyes (40% vs. 13%, p = 0.01). Moreover, the graft-failure eyes experienced significantly more graft-unscrolling/positioning difficulties (40% vs. 10%, p = 0.005), major graft detachment (33% vs. 13%, p = 0.048), and multiple rebubbling (33% vs. 8%, p = 0.04). Women tended to be more likely to experience graft failure than men (87% vs. 64%, p = 0.11). Graft failure tended to associate with younger donor age (70 vs. 75 years, p = 0.06), longer operative times (40 vs. 35 min in graft-success cases, p = 0.07), and thicker preoperative lens thickness (4.78 vs. 4.76 mm, p = 0.10). Other variables did not associate with graft failure, including preoperative BSCVA and ACD and use of triple-DMEK and SF6 (Table 2).

**Table 2:** Univariable analysis of the relationships between graft failure and preoperative, perioperative, and postoperative variables (*n* = 170).

Characteristic	Graft failure (*n* = 15)	Missing values	Graft success (*n* = 155)	Missing values	p*
Patient age, y	71 (60–76)	0	72 (66–78)	0	0.39
Patient female sex	13 (87)	0	100 (64)	0	**0.11**
Operated eyes on right	7 (47)	0	86 (55)	0	0.52
Indication FECD PBK Regraft Post-uveitis	14 (93) 0 (0) 0 (0) 1 (7)	0	145 (94) 5 (3) 5 (3) 0 (0)	0	0.39
Preop AXL, mm	25 (23–25)	0	23 (23–25)	0	**0.02**
<25mm ≥25 mm	9 (60) 6 (40)		135 (87) 20 (13)		**0.01**
Preop ACD, mm	3.22 (2.89–4.32)	0	3.19 (2.84–3.80)	0	0.42
Preop LT, mm	4.78 (4.19–4.79)	10	4.76 (4.58–5.03)	75	**0.10**
Preop BSCVA, logMAR	0.5 (0.4–0.7)	0	0.5 (0.4–0.7)	1	0.24
Donor age, y	70 (62–77)	0	75 (69–82)	0	**0.06**
ECD, cells/mm^2^	2460 (2380–2600)	0	2550 (2400-2680)	0	**0.16**
General anesthesia	15 (100)	0	151 (97)	0	0.99
Triple-DMEK	7 (47)	0	81 (52)	0	0.68
Graft-unscroll/positioning diff	6 (40)	0	16 (10)	0	**0.005**
SF6 use	7 (47)	0	56 (36)	0	0.43
Operative time, min	40 (30–45)	0	35 (30–40)	0	**0.07**
Bubble under iris	0 (0)	0	3 (2)	0	0.99
Major graft detachment	5 (33)	0	20 (13)	0	**0.048**
Any rebubbling	7 (47)	0	49 (31)	0	0.23
No. rebubblings 0 1 ≥2	8 (53) 2 (13) 5 (33)	0	107 (69) 36 (23) 13 (8)	0	**0.04**
Cystoid macular edema	1 (7)	0	12 (8)	0	0.99
Graft rejection	0 (0)	0	0 (0)	0	–

*Generalized linear regression with a random factor on the patient. P-values ≤0.15 are bolded.

ACD, anterior chamber depth; AXL, axial length; BSCVA, best spectacle-corrected visual acuity; diff, difficulties; FECD, Fuchs endothelial corneal dystrophy; LT, lens thickness; PBK, pseudophakic bullous keratopathy; preop, preoperative; SF6, sulfur hexafluoride; y, years.

All eight variables whose p-value in univariable analysis was ≤ 0.15 were then considered for inclusion in the multivariable analysis. However, the eigenvalues and eigenvectors showed that operative time was collinear with graft-unscrolling/positioning difficulties (r = 0.49, p < 0.0001) and rebubbling was collinear with major graft detachment (r = 0.59, p < 0.0001). Being less clinically relevant, operative time and rebubbling were excluded. Lens thickness was also excluded due to unavailable data in the pseudophakic-DMEK cases. Thus, multivariable analysis adjusted for the bilateral cases was conducted with patient sex, preoperative AXL (expressed as <25 and ≥25 mm categories), donor age, graft-unscrolling/positioning difficulties, and major graft detachment. Four of these five variables significantly predicted graft failure, namely, preoperative AXL ≥ 25 mm (OR=5.70, 95%CI = 1.27–25.68, p = 0.02), younger donor age (OR=0.93, 95%CI = 0.88–0.99, p = 0.02), graft-unscrolling/positioning difficulties (OR=6.93, 95%CI = 1.58–30.48, p = 0.01), and major graft detachment (OR=6.28, 95%CI = 1.36–28.96, p = 0.02). The fifth variable, female patient sex, tended to predict graft failure (OR=5.48, 95%CI = 0.76–39.58, p = 0.09). The model accounted for 14% of the total graft-failure variance (R^2^) (Table 3). Similar data were observed when AXL was expressed as a continuous variable (S2 Table).

**Table 3:** Multivariable analysis of factors that predict graft failure (*n* = 170).

Variable	OR	Wald 95% CIs	p*
Patient female sex	5.48	0.76–39.58	0.09
Preoperative AXL ≥ 25 mm†	5.70	1.27–25.68	**0.02**
Donor age	0.93	0.88–0.99	**0.02**
Graft-unscrolling/positioning difficulties	6.93	1.58–30.48	**0.01**
Major graft detachment	6.28	1.36–28.96	**0.02**

*Generalized linear regression with random effects for patients. All independent variables that associated with graft failure on univariable analysis (p < 0.15, see Table 2) were initially considered for inclusion in the multivariable analysis. However, operative time and rebubbling were ultimately excluded due to collinearity with surgical difficulty and major graft detachment, respectively. Lens thickness was also not included because the data for the 82 pseudophakic-DMEK eyes were unavailable.

†Similar data were obtained when preoperative AXL was expressed as a continuous variable (S2 Table).

The model accounted for 14% of total graft-failure variance (R^2^).

AXL, axial length; CI, confidence interval; OR, Odds Ratio.

To assess the effect of excluding the 18 eyes with graft-dissection problems, vitrectomy, missing data, and unusual rejection/infection features from our study cohort, we repeated the multivariable analysis. Very similar results were obtained (S3 Table).

### Posthoc analysis of the relationship between AXL and graft-unscrolling/positioning difficulties

Both preoperative AXL > 25 mm and graft-unscrolling/positioning difficulties were strong predictors of graft failure on multivariable analysis (OR=5.70 and 6.93, respectively; Table 3). This indicates that these variables predict graft failure independently of each other. However, we speculated that longer AXL could also induce graft failure *via* a more indirect pathway, namely, by driving graft-unscrolling/positioning difficulties. This is supported by the fact that longer AXL correlates with deeper ACD [61–63] and deep anterior chambers hamper DMEK-graft unscrolling [14,64–66]. Since multivariable analyses are not necessarily able to detect such indirect mechanisms [67], we tested the notion that longer AXL promotes graft failure by inducing graft-unscrolling/positioning difficulties by assessing the inter-relationships between the four graft-failure predictors. These univariable analyses showed that there was only one significant relationship: eyes with AXL ≥ 25 mm were more than twice as likely to experience graft-unscrolling/positioning difficulties than eyes with AXL < 25 mm (27% vs. 10%, p = 0.0497) (Table 4).

**Table 4:** Univariable relationships between the four covariates that predicted DMEK graft failure (*n* = 170).

Variable	Sex			AXL			Graft-unscrolling/positioning difficulties			Major graft detachment		
	Female *n* = 113	Male *n* = 57	p*	≥25 mm *n* = 26	<25 mm *n* = 144	p*	Yes *n* = 22	No *n* = 148	p*	Yes *n* = 25	No *n* = 145	p*
Patient sex						0.65			0.34			0.99
Female *n* = 113				16 (14)	97 (86)		17 (15)	96 (85)		17 (15)	96 (85)	
Male *n* = 57				10 (18)	47 (82)		5 (9)	52 (91)		8 (14)	49 (86)	
Preoperative AXL			0.65						**0.0497**			0.77
≥25 mm *n* = 26	16 (62)	10 (38)					7 (27)	19 (73)		3 (12)	23 (88)	
<25 mm *n* = 144	97 (67)	47 (33)					15 (10)	129 (90)		22 (15)	122 (85)	
Donor age, y	75 (69-82)	73 (65-82)	0.40	75 (64-79)	75 (69-82)	0.69	76 (67-83)	74 (69-82)	0.81	77 (70-85)	74 (69-81)	0.24
Gr-unscroll/pos diff			0.34			**0.0497**						0.99
Yes *n* = 22	17 (77)	5 (23)		7 (32)	15 (68)					3 (14)	19 (86)	
No *n* = 148	96 (65)	52 (35)		19 (13)	129 (87)					22 (15)	126 (85)	
Major graft detach			0.99			0.77			0.99			
Yes *n* = 25	17 (68)	8 (32)		3 (12)	22 (88)		3 (12)	22 (88)				
No *n* = 145	96 (66)	49 (34)		23 (16)	122 (84)		19 (13)	126 (87)				

The data are expressed as *n* (%). The percentages are expressed according to the variable in the row (e.g., 14% and 18% of females and males had AXL ≥ 25 mm, respectively while 62% and 67% of eyes with AXL ≥ 25 mm were female and male, respectively).

*Generalized linear regression with a random factor on the patient.

AXL, axial length; detach, detachment; Gr-unscroll/pos diff, graft unscrolling/positioning difficulties.

This relationship between preoperative AXL > 25 mm and graft-unscrolling/positioning difficulties was not detected by collinearity assessment with eigenvalues and eigenvectors before multivariable analysis, likely reflecting the modest relationship between these variables. To test that this relationship did not markedly affect the predictive performance of the logistic regression model in Table 3, we excluded either preoperative AXL > 25 mm or graft-unscrolling/positioning difficulties from the multivariable analysis. These posthoc analyses showed that regardless of these exclusions, all significant predictors continued to predict graft failure, including graft-unscrolling/positioning difficulties (OR=8.34, 95%CI = 2.09–33.29, p = 0.004) (S4 Table) and preoperative AXL ≥ 25 mm (OR=6.92, 95%CI = 1.77–27.04, p = 0.01) (S5 Table).

Since operative time and rebubbling were excluded from the multivariable analysis in Table 3 due to collinearity with graft-unscrolling/positioning difficulties (r = 0.49, p < 0.0001) and major graft detachment (r = 0.59, p < 0.0001), respectively, we also asked whether AXL associated with these variables on univariable analysis. Consistent with the collinearity patterns and the univariable association between AXL and graft-unscrolling/positioning difficulties (Table 4), AXL ≥ 25 mm tended to associate with longer operative time (38 vs. 30 min for the AXL ≤ 25 mm subgroup, p = 0.07) but not rebubbling (15% rebubbling rate vs. 12% for the AXL < 25 mm subgroup, p = 0.73).

## Discussion

This study showed that longer preoperative AXL, younger donor age, graft-unscrolling/positioning difficulties, and major graft detachment independently predicted DMEK-graft failure. On univariable analysis, rebubbling also associated significantly with graft failure, reflecting its collinearity with major graft detachment, while longer operative time tended to associate with graft failure, reflecting its collinearity with surgical difficulties. Posthoc univariable analyses suggested that longer AXL could also indirectly induce graft failure by promoting graft-unscrolling/positioning difficulties.

The demographics and indications of our cohort are similar to those in previous studies on factors that shape DMEK-graft failure (S1 Table) [20,26–37]. The DMEK-graft failure rate of our cohort was 9%, consistent with that reported by a Dutch registry study on 752 cases (11%) [20]. BSCVA dropped from 0.5 to 0.08 logMAR in our cohort, similar to the good DMEK visual outcomes reported elsewhere [20,37,68–70].

### Putative mechanisms by which AXL and graft-unscrolling/positioning difficulties promote DMEK-graft failure

Both AXL ≥ 25 mm and graft-unscrolling/positioning difficulties independently predicted graft failure on multivariable analysis (OR=5.70 and 6.93, respectively). However, AXL ≥ 25 mm also associated with more graft-unscrolling/positioning difficulties on posthoc univariable analysis (p = 0.0497). Thus, AXL ≥ 25 mm may promote graft failure (i) indirectly by inducing graft-unscrolling/positioning difficulties and (ii) directly by another mechanism that is independent of graft-unscrolling/positioning difficulties. The putative mechanisms are discussed below.

#### Long AXL may promote graft failure by hampering graft unscrolling.

**Fig 4:**
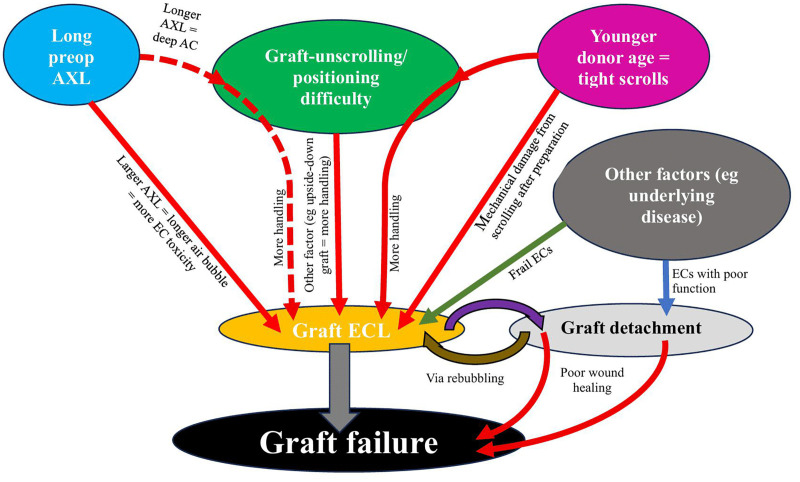
Schematic depiction of potential mechanisms by which pre/perioperative factors could induce DMEK graft failure. The role of ECL is speculative but likely. Solid red arrows indicate hypothetical graft failure-inducing mechanisms. The broken red arrow indicates the indirect AXL-mediated mechanism detected by posthoc univariable analyses. Green and blue arrows indicate how graft disease could generate frail (green arrow) or poorly functioning (blue arrow) endothelial cells. The brown and purple arrows indicate possible mechanisms by which graft detachment could promote graft ECL and *vice versa*. AC, anterior chamber; AXL, axial length; EC, endothelial cell; ECL, endothelial-cell loss.

Fig 4 shows a schematic depiction of the possible mechanisms by which the four predictors could induce graft failure. The hypothesis that long AXL indirectly induces graft failure is depicted by the broken red arrow. This four-part hypothesis proposes that (1) long AXL signifies a more capacious anterior chamber [61–63] that (2) complicates graft unscrolling in the anterior chamber, which (3) increases graft handling that induces extensive ECL, which (4) in turn leads to graft decompensation and failure. With regard to Part 2, it is well known that successful unscrolling with the tapping method requires an optimally shallow anterior chamber. Specifically, in an optimally shallow anterior chamber, the DMEK scroll touches both the cornea and iris. Consequently, on tapping, which generates fluid currents, the scroll not only unfurls, it also remains unfurled. However, in deep anterior chambers, the scroll lacks contact with the iris/cornea and quickly refurls after tapping induces some unscrolling. Notably, a too-shallow anterior chamber also affects unscrolling: the excessive contact of the scroll with the iris/cornea overwhelms the ability of tapping-induced fluid currents to induce unfurling [14,64–66]. Thus, too-deep/long and too-shallow/short eyes can both complicate DMEK-graft unscrolling, requiring the surgeon to generate an optimally shallow anterior chamber by adding or removing fluid during the unscrolling procedure. The effect of too-shallow/short eyes is supported by Hayashi et al., who reported that DMEK-graft unscrolling is often hindered in Asian eyes, which generally have shallow anterior chambers and shorter AXL [71]. Our study suggests the converse may also be true, namely, that too-deep/long eyes also complicate DMEK-graft unscrolling.

Our hypothesis also states that graft-unscrolling difficulties induce ECL (Part 3), which leads to graft failure (Part 4). Part 3 is also supported by Hayashi et al.: they reported that too-shallow/short eyes (which associate with graft-unscrolling difficulty) display greater graft ECL [71]. Borroni et al. also observed that shorter AXL associates with more post-DMEK ECL [51]. Part 4 is supported by Çakmak et al., who found that shallow eyes have a 3-fold higher risk of graft failure after DMEK [72]. Multiple studies also report that unscrolling/positioning difficulties associate with ECL [10,11,13,20,73–75] and graft failure [75]. Thus, the literature indirectly supports the notion that not only too-shallow/short eyes but also eyes with very large AXL (≥25 mm) can promote graft-unscrolling difficulties, which induce ECL, which in turn promotes graft failure. Notably, this effect of too-large eyes may be compounded by the fact that it is difficult to generate an optimally shallow anterior chamber in such eyes [14].

Our finding suggests that eyes with AXL ≥ 25 mm should perhaps be treated with DSAEK rather than DMEK. Although the visual outcomes after DSAEK are not as good as that of DMEK, DSAEK is much less prone to graft detachment and easier to manage postoperatively [9]. Alternatively, we can consider using techniques that help induce shallow ACDs in post-vitrectomy eyes (which lack posterior pressure) and eyes with vitreous syneresis, previous PKP, or glaucoma shunts (which can be difficult to shallow). These techniques include denting the bulbar surface with a finger or surgical instrument [76] or temporarily placing cellulose sponges between the bulbar surface and the eyelid speculum in a hands-free manner [14]. Insertion of a transcleral infusion cannula to artificially increase vitreous pressure could also be considered. Moreover, it is important to ensure that patients with large anterior chambers remain supine after DMEK: Bennett et al. showed that while postoperative positioning has little effect on the amount of graft coverage by the endotamponade when the ACD is small, large ACDs associate with worse coverage, especially in pseudophakic eyes [77].

#### Long AXL also induces graft failure independently of graft-unscrolling difficulties.

Our multivariable analyses showed that longer AXL also directly induces graft failure (Fig 4, solid left-hand red arrow). The underlying mechanism could involve the larger volume of air/gas needed for endotamponade in eyes with longer AXL [61–63]: air is inherently toxic to the corneal endothelium [78,79], and the bubble itself may induce mechanical damage [80,81] or hamper endothelial-cell permeability or access to the aqueous humor and nutrients [82,83]. Since a larger bubble takes longer to disappear, this would extend bubble contact with the endothelium, thus potentially enhancing ECL that in turn promotes graft failure [22–24].

#### Graft-unscrolling/positioning difficulties could induce graft failure independently of AXL.

Our analyses also suggest that graft-unscrolling/positioning difficulty, the largest independent predictor of graft failure, plays an AXL-independent role in graft failure (Fig 4, middle solid red arrow). This may reflect the fact that this variable includes graft-positioning difficulties that require additional graft handling that exacerbates ECL. As mentioned above, multiple studies show that increased graft handling associates with greater ECL [10–12,20,75].

Note that we could not test whether long AXL or graft-unscrolling/positioning difficulties did in fact induce greater ECL, because the eyes whose DMEK graft eventually failed were edematous, which hindered postoperative ECD measurements.

#### Role of anterior chamber depth.

Although ACD correlates with AXL [61–63], we did not find that preoperative ACD associated with graft failure on univariable analysis. This may reflect the fact that (i) 48% of our eyes were pseudophakic at the time of DMEK, and (ii) preoperative ACD measurements with IOLMaster 700 are not reliable in pseudophakic eyes because the IOL and natural lens have different optical properties [84]. By contrast, IOLMaster measures preoperative AXL almost as well in pseudophakic eyes as in phakic eyes [85]. Univariable analysis of the 88 triple-DMEK eyes in our cohort did not show an association between ACD and graft failure, but this may reflect the small number of graft failures in this group (*n =* 7).

### Relationship between major graft detachment and graft failure

We observed that major graft detachment that required rebubbling also strongly predicted DMEK-graft failure (OR=6.28). This association has been observed previously (S1 Table) [20]. Graft detachment likely reflects poor engagement of the wound-healing processes that integrate the donor Descemet membrane with the recipient stroma. Any factor that impairs the orderly and timely progression of this wound-healing cascade can thus result in graft detachment once the endotamponade wears off. These factors could include new edema or failure to clear existing edema after DMEK: such fluid disrupts the stromal microarchitecture and intercellular communications needed for proper wound healing [86–89]. A major cause of this edema may be graft ECL during surgery (Fig 4, purple arrow) that is generated, for example, by variables such as long AXL. Post-DMEK edema could also reflect preexisting endothelial-cell frailty (Fig 4, green arrow) or functional incompetence (Fig 4, blue arrow) that was not detected by the eye bank (Fig 3, dark grey oval).

Notably, rebubbling, the treatment for graft detachment, may itself promote ECL, thus worsening outcomes (Fig 4, brown arrow): multiple rebubbling associated with graft failure on our univariable analysis (p = 0.04) and other studies show that rebubbling, especially multiple rebubbling, associates with more ECL [21,32,40,90,91]. However, since DMEK grafts undergoing detachment may already have more ECL than attached grafts [92], it is unclear whether rebubbling does in fact induce additional ECL.

Graft detachment itself does not induce ECL: 2–5 weeks after complete detachment, DMEK grafts floating in the anterior chamber continue to have high ECDs [47]. Thus, the relationship between graft detachment and graft failure likely reflects factors that promote endothelial incompetence, which leads to wound-healing failure, graft detachment, and ultimately graft failure (Fig 4, bottom right red arrows).

### Relationship between donor age and graft failure

We found that grafts from younger donors weakly predicted more DMEK-graft failure (OR=0.93, 95%CI = 0.88–0.99, p = 0.02), consistent with previous multivariable [93] and univariable [29] analyses showing that younger donor age predicts more graft detachment/rebubbling. Several studies suggest that this reflects the fact that older DMEK grafts are thicker and less elastic than younger grafts, which lead to looser scrolls [13,94]: it was proposed that these loose scrolls result in shorter graft-unscrolling times and less handling, which minimizes postoperative ECL [13] (Fig 4, curving top right red arrow). It should be noted, however, that graft-unscrolling/positioning difficulties did not associate with graft-donor age in our univariable analyses (Table 4). An alternative possibility is that graft scrolling inflicts detrimental ECL-inducing mechanical forces on the endothelial cells before transplantation (Fig. 3, straight top right red arrow). For example, the tight scrolling of young grafts (with endothelium on the outside [95]) could compress and rub the cells on the inside layers. These mechanical forces may both damage the cells physically and initiate detrimental mechanosignalling in the endothelial cells that induces their death [96].

However, it should be emphasized that the effect of donor age in our study was mild (OR=0.93) and the median ages of the patients who did and did not undergo graft failure were 70 and 75 years, respectively. Moreover, many other studies have not observed a link between graft-donor age and graft failure itself (S1 Table) [20,28,30,31,36,37] or its surrogates, namely, rebubbling, graft detachment [20,30,37,42,97–102], and ECL [10,11,18,54,93,97]. Thus, the clinical relevance of this finding may be limited.

### Relationship between other variables and graft failure

We observed that women tended to undergo graft failure more often than men on both univariable (p = 0.09) and multivariable (OR=5.48, 95%CI = 0.76–39.58, p = 0.09) analysis. However, many other studies did not observe a relationship between patient sex and graft failure [31,20], rebubbling/graft detachment [20,41,69,93,98–100,103], or ECL [11,17,18,53,54,93,104]. The clinical relevance of this variable is likely to be low.

Patient age did not affect DMEK-graft failure, similar to several other studies [30,32]. This was also true for lens thickness. Similarly, preoperative visual acuity did not associate with DMEK-graft survival in our study or with graft detachment [69], rebubbling [19], or ECL [10,17,53,104] in other studies. Triple-DMEK did not increase graft failure in our or two other graft-failure studies [20,35] but one study found a univariable association (p = 0.03) [34] (S1 Table). Several studies [38,39,43,99,102,105] but not others [10,20,34,35,37,41,54,69,70,98,100,101,104,105–107] also reported that triple-DMEK associates with more rebubbling, graft detachment, and/or ECL. Interstudy variation in the inclusion of phakic-DMEK cases, which associate with less rebubbling than triple-DMEK and pseudophakic-DMEK [43,102,99], may partly explain these discrepancies.

Our univariable analysis and two other studies [27,28] found that using SF6 instead of air for endotamponade did not alter graft-failure rates (S1 Table). However, other studies suggest SF6 prevents graft detachment and rebubbling [28,68,108–112] while not altering ECL [18,53,113]. Further studies assessing the ability of SF6 to improve DMEK-graft survival are needed.

### Study limitations

This study has several limitations. First, its retrospective design may have introduced selection and information bias, although the data were recorded prospectively. Second, as a monocentric study, it has limited generalizability. However, since all surgeries were performed by a single experienced surgeon, this may have minimized confounding related to variations in surgical technique. Third, all DMEKs were conducted as described by Melles [2], namely, with surgeon-prepared grafts rather than eyebank-prepared grafts, which are used in some countries. However, several studies have shown that graft preparation technique does not affect DMEK outcomes [38,114,115]. Fourth, the sample size was relatively small (*n* = 170). Fifth, the number of graft failure cases (*n =* 15) was small. Our study findings must be corroborated by larger scale studies. Sixth, 94% of our patients had FECD. Thus, it is unclear whether our findings can be extrapolated to other DMEK indications such as BK or regraft. Seventh, while ECL may play a central role in graft failure, we could not confirm that it participated in the mechanisms proposed in Fig 4 because postoperative ECD in failing grafts cannot be reliably measured by specular microscopy. Studies with more accurate methods such as confocal microscopy [116] are needed. Eighth, we did not measure other variables that could support the proposed mechanisms in Fig 4, including endotamponade duration and scroll width. Ninth, the individual contributions of graft-unscrolling difficulty and graft-positioning difficulty to graft failure could not be explored because most problematic cases had both. Tenth, the ability of multivariable analyses to detect factors that cause the outcome depends on the selected covariates, their inter-relationships, the study population, and measurement accuracy [67]. While we tested 20 covariates, we did not examine the contributions of other variables that may promote DMEK-graft failure, including surgeon learning curve [117], graft marking [118], graft storage conditions [93,119], donor diabetes [120,121], postoperative intraocular pressure changes [122], and glaucoma [123]. Thus, our multivariable analysis results, including the effect of long AXL, require validation by other groups.

## Conclusions

Our multivariable analysis showed that AXL, which has been rarely studied in DMEK, predicted graft failure. Specifically, AXL ≥ 25 mm predicted significantly more graft failure than shorter AXL. Thus, if a patient has AXL ≥ 25 mm, it may be necessary to consider using DSAEK, which has worse visual outcomes than DMEK but is easier to conduct. Alternatively, mitigating surgical approaches should be taken to ensure adequate anterior-chamber shallowing during DMEK in these eyes. Additional studies are needed to confirm our findings and determine the relative merits of DSAEK and DMEK in patients with high myopia. DMEK-graft failure may also be reduced by minimizing graft-unscrolling/positioning difficulties. Assiduously treating major graft detachment with rebubbling could also improve graft survival, although it is also possible that rebubbling, especially multiple rebubbling, inflicts counterproductive ECL. Studies with techniques that can measure ECD in edematous grafts are needed to determine the pros and cons of DMEK-graft rebubbling.

## Supporting information

S1 Table Summary of the literature searching for risk factors for graft failure after DMEK. (DOCX)

S2 Table Multivariable analysis of factors that predict graft failure, with axial length expressed as a continuous variable (*n* = 170). (DOCX)

S3 Table Multivariable analysis of factors that predict graft failure in all consecutive eyes before exclusions (*n* = 188). (DOCX)

S4 Table Posthoc multivariable analysis of factors that predict graft failure with axial length excluded (*n* = 170). (DOCX)

S5 Table Posthoc multivariable analysis of factors that predict graft failure with graft-unscrolling/positi*n*oning difficulty excluded (*n* = 170). (DOCX)
