# Supplementary material for: Preoperative and perioperative factors that predict graft failure 1 year after Descemet membrane endothelial keratoplasty
Source: PLoS One. 2026 Jul 24;21(7):e0352687. doi: 10.1371/journal.pone.0352687 (PMC13399445; doi:10.1371/journal.pone.0352687)
Supplement: S1 Table — (DOCX) [file pone.0352687.s005.docx]

## SUPPLEMENTARY TABLE S5. Posthoc Multivariable Analysis of Factors That Predict Graft Failure, With Graft-Unscrolling/Positioning Difficulties Excluded (*n*=170)

| Characteristic | OR | Wald 95% CIs | p* |
| --- | --- | --- | --- |
| Patient female sex | 5.46 | 0.91–32.72 | 0.06 |
| Preop axial length ≥25 mm | 6.92 | 1.77–27.04 | **0.01** |
| Donor age | 0.95 | 0.90–0.99 | **0.03** |
| Graft-unscroll/position diff | XXX | XXX | XXX |
| Major graft detachment | 4.77 | 1.19–19.23 | **0.03** |

*Generalized linear regression with random effects for patients.

CI, confidence interval; diff, difficulties; OR, Odds ratio; preop, preoperative.
