# Supplementary material for: Preoperative and perioperative factors that predict graft failure 1 year after Descemet membrane endothelial keratoplasty
Source: PLoS One. 2026 Jul 24;21(7):e0352687. doi: 10.1371/journal.pone.0352687 (PMC13399445; doi:10.1371/journal.pone.0352687)
Supplement: S2 Table — (DOCX) [file pone.0352687.s001.docx]

## S1 TABLE. Summary of the Literature Searching for Risk Factors for Graft Failure After DMEK

| **Author year** | **Type study** | **No. eyes** | **Age** | **Female Sex %** | **% FECD** | **Timepoint & frequency GF†** | **Type of analysis** | **Older pt age** | **Female sex** | **Older donor age** | **Indication** | **Triple-DMEK** | **Surgical difficulties** | **SF6 use** | **Preop. VA** | **Graft detach** | **Rebubbling** |
| --- | --- | --- | --- | --- | --- | --- | --- | --- | --- | --- | --- | --- | --- | --- | --- | --- | --- |
| Our study | Re | 171 | 72 | 66 | 94 | 3mo 9% | UV | NS | 0.09 | 0.10 | NS | NS | 0.0002 | NS | NS | 0.048 | #RBs 0.02 |
|  |  |  |  |  |  |  | MV |  | 0.09 | 0.01 |  |  | 0.0004 | NS |  | 0.005 |  |
| Fu 2023[75] | Re | 176 | 71 | 58 | 88 | 5y 5% | MV |  |  |  |  |  | HR1.8, incl diff unfold |  |  |  |  |
| Maier 2023[26] | Re | 150 all glauc | 72 | - | 49 | 3y 17% | UV |  |  |  | 0.004 |  |  |  |  |  | 0.01 |
|  |  |  |  |  |  |  | MV |  |  |  | HR 9 |  |  |  |  |  | NS |
| Tutas 2022[30] | Re | 114 | 70 | - | 36 | 3y 10% | UV | NS |  | NS | 0.092 |  |  |  |  |  |  |
|  |  |  |  |  |  |  | MV |  |  | NS | Sig |  |  |  |  |  |  |
| Fliotsos 2022[31] | Re | 10,  598 | 69 | 61 | 72 | 2mo 1% | UV | NS | NS | NS | 0.01 |  |  |  |  |  |  |
|  |  |  |  |  |  |  | MV | 0.06 |  | NS | NS |  |  |  |  |  |  |
| Dunker 2021[20] | Pr | 752* | 71 | 53 | 90 | 3mo GF 11% | UV | 0.04 | NS | NS | NS | NS | 0.001, incl diff unfold |  |  |  |  |
|  |  |  |  |  |  |  | MV | NS | NS | NS | NS | NS | 0.004, incl diff unfold |  |  |  | 0.01 |
| Gund 2021[32] | Re | 463 | 71 | - | 85 | 2y 4% | MV | NS |  |  |  |  |  |  |  |  | NS #RBs |
| Achir 2021[33] | Re | 167 | 76 | - | - | Youngest v oldest qrtl  2y GF 5% v 41% | UV | 0.006 |  |  |  |  |  |  |  |  |  |
|  |  |  |  |  |  |  | MV | HR 17 |  |  |  |  |  |  |  |  |  |
| Fajar 2021[34] | Re | 329 | 72 | 67 | 73 | 1y GF 17% | UV |  |  |  |  | 0.03 |  |  |  |  |  |
| Rickm 2020[35] | Re | 120 | - | - | 80-88 | 6mo 8% | UV |  |  |  | NS | NS | NS diff unfold |  |  |  |  |
| Schau 2020[36] | Re | 1765 | - | 57 | 87 | 3y 3% | UV |  |  | NS |  |  |  |  |  |  |  |
| Godin 2019[37] | Re | 139* | 66 | 69 | 98 | 1y 4% | UV |  |  | NS |  |  |  |  |  |  |  |
| Rickma 2018[27] | Re | 108 | 70, 72 | - | 100 | SF6 v air  6mo GF: 6% v 4% | UV |  |  |  |  |  |  | NS |  |  |  |
| Schau 2017[28] | Re | 854 | 70 | 57 | 87 | 1y 2% | UV |  |  |  |  |  |  | NS |  |  |  |
| Schaub 2016[29] | Re | 529* | 66 | 78 | 86 | Young v old donors, 1y GF both 3.2% | UV |  |  | NS |  |  |  |  |  |  |  |

* Second eyes included in UV or MV analysis without statistical correction for non-independence of samples.

† Graft failure was defined as any graft failure in the indicated period, including that caused by graft rejection.

#RBs, number of rebubbling sessions; detach, detachment; diff unfold, difficulties unfolding the graft; DMEK, Descemet-membrane endothelial keratoplasty; FECD, Fuchs endothelial corneal dystrophy; GF, graft failure; glauc, glaucoma; HR, Hazard ratio; incl diff unfold, the variable ‘surgical difficulties’ included difficulties unscrolling the graft; mo, month; MV, multivariable; NS, not significant; preop., preoperative; Pr, prospective; qrtl, quartile; Re, retrospective; SF6, sulfur hexofluoride; Sig, significant; UV, univariable; v, versus; VA, visual acuity; y, year.
