# Supplementary material for: Preoperative and perioperative factors that predict graft failure 1 year after Descemet membrane endothelial keratoplasty
Source: PLoS One. 2026 Jul 24;21(7):e0352687. doi: 10.1371/journal.pone.0352687 (PMC13399445; doi:10.1371/journal.pone.0352687)
Supplement: S3 Table — (DOCX) [file pone.0352687.s002.docx]

## SUPPLEMENTARY TABLE S2. Multivariable Analysis of Factors That Predict Graft Failure, With Axial Length Expressed as a Continuous Variable (*n*=170)

| Characteristic | OR | Wald 95% confidence interval | P* |
| --- | --- | --- | --- |
| Patient female sex | 4.39 | 0.70–27.47 | 0.11 |
| Preop axial length, mm | 1.58 | 1.07–2.33 | **0.02** |
| Donor age | 0.93 | 0.88–0.98 | **0.01** |
| Graft-unscroll/position difficulties | 7.87 | 1.82–34.01 | **0.01** |
| Major graft detachment | 6.50 | 1.39–30.39 | **0.02** |

*Generalized linear regression with random effects for patients.

AXL, axial length; CI, confidence interval; OR, Odds Ratio; preop, preoperative.
