# Supplementary material for: Preoperative and perioperative factors that predict graft failure 1 year after Descemet membrane endothelial keratoplasty
Source: PLoS One. 2026 Jul 24;21(7):e0352687. doi: 10.1371/journal.pone.0352687 (PMC13399445; doi:10.1371/journal.pone.0352687)
Supplement: S4 Table — (DOCX) [file pone.0352687.s003.docx]

## SUPPLEMENTARY TABLE S3. Multivariable Analysis of Factors That Predict Graft Failure in All Consectutive Eyes Before Exclusions (*n*=188)

| Characteristic | OR | Wald 95% confidence interval | P* |
| --- | --- | --- | --- |
| Patient female sex | 2.55 | 0.59–11.03 | 0.21 |
| Preop axial length, mm | 4.66 | 1.23–17.67 | **0.02** |
| Donor age | 0.94 | 0.90–0.99 | **0.02** |
| Graft-unscroll/position difficulties | 6.07 | 1.63–22.66 | **0.01** |
| Major graft detachment | 5.45 | 1.43–20.87 | **0.01** |

*Generalized linear regression with random effects for patients.

AXL, axial length; CI, confidence interval; OR, Odds Ratio; preop, preoperative.
