# Supplementary material for: Preoperative and perioperative factors that predict graft failure 1 year after Descemet membrane endothelial keratoplasty
Source: PLoS One. 2026 Jul 24;21(7):e0352687. doi: 10.1371/journal.pone.0352687 (PMC13399445; doi:10.1371/journal.pone.0352687)
Supplement: S5 Table — (DOCX) [file pone.0352687.s004.docx]

## SUPPLEMENTARY TABLE S4. Posthoc Multivariable Analysis of Factors That Predict Graft Failure, With Axial Length Excluded (*n*=170)

| Characteristic | OR | Wald 95% CIs | p* |
| --- | --- | --- | --- |
| Patient female sex | 3.52 | 0.81–33.66 | 0.14 |
| Preop axial length ≥25 mm | XXX | XXX | XXX |
| Donor age | 0.94 | 0.89–0.99 | **0.02** |
| Graft-unscroll/position diff | 8.34 | 2.09–33.29 | **0.004** |
| Major graft detachment | 5.20 | 1.24–21.82 | **0.03** |

*Generalized linear regression with random effects for patients.

CI, confidence interval; diff, difficulties; OR, Odds ratio; preop, preoperative.
