# Supplementary material for: Preoperative and perioperative factors that predict graft failure 1 year after Descemet membrane endothelial keratoplasty
Source: PLoS One. 2026 Jul 24;21(7):e0352687. doi: 10.1371/journal.pone.0352687 (PMC13399445; doi:10.1371/journal.pone.0352687)
Supplement: Attachment — Submitted filename: Response to Reviewers.docx [file pone.0352687.s007.docx]

**Response to Reviewers**

# Editor comments

1. Please ensure that your manuscript meets PLOS ONE's style requirements, including those for file naming. The PLOS ONE style templates can be found at

<https://journals.plos.org/plosone/s/file?id=wjVg/PLOSOne_formatting_sample_main_body.pdf> and

<https://journals.plos.org/plosone/s/file?id=ba62/PLOSOne_formatting_sample_title_authors_affiliations.pdf>

**Reply:** We ensured during revision that the paper conformed to these style requirements.

2. We note that you have indicated that there are restrictions to data sharing for this study. For studies involving human research participant data or other sensitive data, we encourage authors to share de-identified or anonymized data. However, when data cannot be publicly shared for ethical reasons, we allow authors to make their data sets available upon request. For information on unacceptable data access restrictions, please see <http://journals.plos.org/plosone/s/data-availability#loc-unacceptable-data-access-restrictions>.

Before we proceed with your manuscript, please address the following prompts:

a) If there are ethical or legal restrictions on sharing a de-identified data set, please explain them in detail (e.g., data contain potentially identifying or sensitive patient information, data are owned by a third-party organization, etc.) and who has imposed them (e.g., a Research Ethics Committee or Institutional Review Board, etc.). Please also provide contact information for a data access committee, ethics committee, or other institutional body to which data requests may be sent.

Please update your Data Availability statement in the submission form accordingly.

**Reply:** In France, the dataset cannot be shared on a repository, even when the patients have been anonymized. Legally, we cannot add to or change the original Data Availability statement, which is:

“*The datasets generated during and/or analyzed during the current study are not publicly available according to French Law No. 2018-493 of June 20, 2018 on the protection of personal data (The General Data Protection Regulation (Regulation (EU) 2016/679) (GDPR: article 9) but are available from the Clinical Research Support Platform (Plateforme d’Appui à la Recherche Clinique [PARC]) of the Regional Central Hospital (CHR) of Metz-Thionville on reasonable request (email: projetrechercheclinique@chr-metz-thionville.fr, tel: +33 3 87 17 98 82). All nonarchived data is subject to daily backups while all archived data is subject to duplicate storage at two different sites. This data processing is compliant with a baseline reference methodology (MR001) to which the CHR MetzThionville signed a compliance commitment on October 8, 2018.*”

# Reviewer #1:

The authors present a retrospective analysis of factors associated with graft failure after DMEK. The following points would be a starting point to improve the manuscript:

**Reply:** Thank you very much for the time you have taken to review our manuscript, and for your helpful comments. We feel the manuscript is much improved after revision according to these and the other reviewer comments.

## Comment 1:

Was superficial keratectomy carried out for any cases? Please report.

**Reply:** No, we did not perform any superficial keratectomies.

To address this point, we added the variable “Superficial keratectomy”and the fact that none were performed to Table 1, and the following text to the manuscript body:

Methods, Surgical techniques, Lines 189-191: “*If the recipient eye had concomitant anterior corneal surface pathology, superficial keratectomy was conducted with alcohol delamination, followed by polishing with a cellulose sponge and irrigation.*”

## Comment 2:

Graft variables are not sufficiently reported and could also influence graft survival. Please comment on donor age, storage duration, and endothelial cell density in greater detail.

**Reply:** We have addressed this comment about each of these three variables as follows.

(i) **Donor age:** the donor age of our grafts was shown in Table 1, and Tables 3–4 indicate that indeed, younger donor age increases graft failure (Results, Lines 335, 349). This may reflect the greater handling involved with the scroll-prone grafts from younger donors and/or scrolling of the graft after preparation. These points are detailed in the Discussion (Line 512-526).

To address this comment, we expanded our description of the cohort:

Results, Preoperative and operative characteristics, Line 312: “*Median graft donor age was 74 years*.”

(ii) **Graft-storage duration**: we unfortunately found it difficult to retrieve these data for our study and were therefore not able to assess its role. However, existing studies on DMEK grafts that were stored in organ culture suggest that graft-storage duration is unlikely to markedly affect DMEK graft failure:

- Univariable analyses found that duration of DMEK-graft storage in organ culture does not significantly affect early DMEK graft failure^20^ or rebubbling^20,16^ rates.
- A multivariable analysis also showed that graft-storage time did not predict detachment rates.^96^
- While two multivariable analyses did show that 6-month^96^ and 5-year^123^ ECD after DMEK drops mildly by 0.7% and 1.2% per extra day in organ culture, respectively, another univariable analysis did not find this relationship 2 years after DMEK.^17^

^20^ Dunker et al. Br J Ophthalmol. 2021. doi:10.1136/bjophthalmol-2020-317041

^16^ Gerber-Hollbach et al. Cornea. 2017. doi:10.1097/ICO.0000000000001220

^96^ Rodríguez-Calvo De Mora et al. JAMA Ophthalmol. 2016. doi:10.1001/jamaophthalmol.2015.4499

^123^ Birbal et al. Cornea. 2020. doi:10.1097/ICO.0000000000002120

^17^ Peraza-Nieveset al. Cornea. 2017. doi:10.1097/ICO.0000000000001176

Thus, it seems that graft-storage duration has at best relatively mild effects on DMEK graft-success rates. To address this point, we added a new reference (Birbal et al., 2020, ref. 123):

Study Limitations, Line 578: “*While we tested 20 covariates, we did not examine the contributions of other variables that may promote DMEK-graft failure, including surgeon learning curve [121], graft marking [122], graft storage conditions [96,123]...*”

(iii) **Endothelial cell density (ECD)**: all of our grafts had an ample preoperative ECD (all >2200 cells/mm^2^). Nonetheless, to address this point, we examined the effect of preoperative ECD on graft failure by conducting an additional univariable analysis. This showed that the median (interquartile range) preoperative ECD of the grafts that did and did not fail in the first postoperative year was 2460 (2380–2600) and 2550 (2400–2680) cells/mm^2^, respectively and the two groups did not differ significantly in terms of preoperative ECD (p=0.16).

To address this point, we added these ECD data to our analyses. This changed the number of pre/perioperative independent variables we studied from 19 to 20 (see Lines 43, 122, 259, 579). Tables 1 and 2 now include the preoperative ECD data. We also added the following texts to the revised manuscript:

Methods, Preoperative variables, Lines 161-163: “*Preoperative graft ECD was determined by manual counting under an optic microscope by an eye-bank technician followed by a recount by another technician.*”

Methods, Surgical techniques, Lines 175-180: “*These preoperative ECD measurements were made just before transport, which occurred 3–4 weeks after button harvest. For transport, the buttons were placed in dextran-containing transport medium (CorneaJet; Eurobio) and shipped to our hospital the day before surgery. They were exposed to the transport medium for 3 days. DMEK grafts were generated from the corneoscleral buttons immediately before surgery.*”

## Comment 3:

Younger age is poorly defined in the study. Clarify the definition and quantify in the results. 70 vs. 75 is mentioned at line 321, but that age difference does not seem clinically significant.

**Reply:** We agree and have reduced and revised the Abstract and Discussion to reflect this. The following texts were deleted from the Abstract and Discussion:

Abstract: “*Younger donor age weakly predicted DMEK-graft failure, possibly due to tighter scrolling of younger grafts.*”

Discussion, relationship with donor age: “*Nonetheless, our finding suggests that as long as they have ample endothelial-cell reserve, older donors may be preferrable to younger donors for DMEK, particularly if the surgeon is still in training* [9]*.*”

The remaining Discussion text was modulated:

Discussion, Relationship with donor age, Lines 522-535: “*It should be noted, however, that graft-unscrolling/positioning difficulties did not associate with graft-donor age in our univariable analyses (Table 4). An alternative possibility is that graft scrolling inflicts detrimental ECL-inducing mechanical forces on the endothelial cells before transplantation (Fig. 3, straight top right red arrow). For example, the tight scrolling of young grafts (with endothelium on the outside [98]) could compress and rub the cells on the inside layers. These mechanical forces may both damage the cells physically and initiate detrimental mechanosignalling in the endothelial cells that induces their death [99].*

*However, it should be emphasized that the effect of donor age in our study was mild (OR=0.93) and the median ages of the patients who did and did not undergo graft failure were 70 and 75 years, respectively. Moreover, many other studies have not observed a link between graft-donor age and graft failure itself (S1 Table) [20,28,30,31,36,37] or its surrogates, namely, rebubbling, graft detachment [20,30,37,42,100–105], and ECL [10,11,18,54,96,100]. Thus, the clinical relevance of this finding may be limited.”*

## Comment 4:

While certainly a valid technique, suggesting DSAEK as an alternative in eyes with AL is not supported directly by the study. Would suggest to avoid specifically suggesting alternate technique without comparative data supporting this.

**Reply:** We believe that identifying this problem of long AXL requires us to suggest alternative strategies to improve outcomes. We are currently conducting DSAEK in eyes with AXL≥25 that are otherwise eligible for DMEK, and will publish the outcomes of these cases in due course. However, we agree that we should reduce the emphasis on DSAEK as an alternative to DMEK in AXL≥25 mm cases.

To address this comment, we removed the following text from the Abstract:

“*DSAEK may be more appropriate for such eyes.*”

## Comment 5:

The timeline for graft failure would be useful to know (what % of grafts failed at 3 months vs. 6 months. vs. later). Suggestion to include survival plot.

**Reply:** Thank you for this comment. To address it, we added the following texts to the Statistics and Results sections and created a new Fig 3 to show the graft survival curve (the original Fig 3 is now designated Fig 4). We also added/clarified details regarding follow-up of the cohort:

Abstract, Lines 40-42: “*This retrospective cohort study included consecutive eyes that underwent DMEK in 2015-2023 in a regional referral hospital and were followed for at least 12 months. DMEK-graft failure was defined as need for regrafting during follow-up.*”

Abstract, Lines 49-50: “*Median (range) follow-up was 24 (12–29) months, during which 15 grafts (9%) failed.*”

Methods, Surgical techniques, Lines 235–237: “*If non-adhesion continued despite up to four rebubblings, corneal edema persisted after 3 months, and/or new corneal edema arose during follow-up, the graft was considered to have failed*”

Methods, Collected variables, Lines 250-251: “*Graft failure was defined as need for a new graft during follow-up due to any reason*”

Statistics, Lines 273-274: “*To depict the time course of graft failures, Kaplan-Meier survival analysis was conducted.*”

Results, Lines 302–303: “*The median (IQR; range) follow-up duration of these 170 eyes was 24 (12–24; 12–29) months.*”

Results, Postoperative outcomes, Lines 322-326: “*During follow-up, 15 (9%) of the eyes had experienced graft failure (Table 1).* *Of these 15 grafts, seven (47%), three (20%), two (13%), and three (20%) failed at ≤3 months, 4–6 months, 7–12 months, and >12 months, respectively. The median (IQR) duration to graft failure was 5 (2–9) months. Fig 3 shows the survival curve of all grafts.*”

Figure legends, Lines 1034-1037:

“*Fig 3. DMEK graft survival curve.*

*Fig 4.* ***Schematic depiction of potential mechanisms by which pre/perioperative factors could induce DMEK graft failure***.”

# Reviewer #2:

Dear Authors,

thank you for this interesting and relevant manuscript. From my perspective, it would strengthen the study to include all patients—including those who underwent vitrectomy and those with suboptimal graft conditions—in the primary analysis to better reflect real-world clinical practice. A subsequent subgroup analysis excluding these cases could then provide additional insight into outcomes under more ideal conditions.

Such an approach would allow the reader to better appreciate both the “real-life” effectiveness and the results under optimized circumstances.

Kind regards!

**Reply:** Thank you very much for your time and helpful suggestion.

To address this comment, we repeated the multivariable analysis with all 188 eyes, including the 17 eyes with graft-dissection problems, the single eye with vitrectomy, and the four eyes with missing AXL and/or ACD values. Almost exactly the same results were obtained.

To address this, we added the following texts and new S3 Table (the original S3 and S4 Tables are now designated S4 and S5 Tables, respectively, see revised Supplementary Data):

Methods, Patient selection, Lines 152-153: “*To assess the effect of these exclusions on our findings, we repeated our multivariable analysis with all consecutive patients*.”

Statistics, Lines 274-277: “*To assess the effect of excluding cases with graft-dissection problems, vitrectomy, missing data, and complicated rejection/infection features on our multivariable-analysis findings, we repeated this analysis with all consecutive patients.*”

Results, Multivariable analyses, Lines 359-361: “*To assess the effect of excluding the 18 eyes with graft-dissection problems, vitrectomy, missing data, and unusual rejection/infection features from our study cohort, we repeated the multivariable analysis. Very similar results were obtained (S3 Table).*”
