# Supplementary material for: Preoperative and perioperative factors that predict graft failure 1 year after Descemet membrane endothelial keratoplasty
Source: PLoS One. 2026 Jul 24;21(7):e0352687. doi: 10.1371/journal.pone.0352687 (PMC13399445; doi:10.1371/journal.pone.0352687)
Supplement: Peer review history

# Peer review: Decision Letter 0

**DOI:** 10.1371/journal.pone.0352687.r001

**Authors:** Rajiv R. Mohan

29 Mar 2026

PONE-D-26-11203

Preoperative and perioperative factors that predict graft failure 1 year after Descemet membrane endothelial keratoplasty

PLOS One

Dear Dr. Perone,

Thank you for submitting your manuscript to PLOS ONE. After careful consideration, we feel that it has merit but does not fully meet PLOS ONE’s publication criteria as it currently stands. Therefore, we invite you to submit a revised version of the manuscript that addresses the points raised during the review process.

Please submit your revised manuscript by May 13 2026 11:59PM. If you will need more time than this to complete your revisions, please reply to this message or contact the journal office at plosone@plos.org. When you're ready to submit your revision, log on to https://www.editorialmanager.com/pone/ and select the 'Submissions Needing Revision' folder to locate your manuscript file.

Please include the following items when submitting your revised manuscript:

- A letter that responds to each point raised by the academic editor and reviewer(s). You should upload this letter as a separate file labeled 'Response to Reviewers'.
- A marked-up copy of your manuscript that highlights changes made to the original version. You should upload this as a separate file labeled 'Revised Manuscript with Track Changes'.
- An unmarked version of your revised paper without tracked changes. You should upload this as a separate file labeled 'Manuscript'.

If you would like to make changes to your financial disclosure, please include your updated statement in your cover letter. Guidelines for resubmitting your figure files are available below the reviewer comments at the end of this letter.

If applicable, we recommend that you deposit your laboratory protocols in protocols.io to enhance the reproducibility of your results. Protocols.io assigns your protocol its own identifier (DOI) so that it can be cited independently in the future. For instructions see: https://journals.plos.org/plosone/s/submission-guidelines#loc-laboratory-protocols. Additionally, PLOS ONE offers an option for publishing peer-reviewed Lab Protocol articles, which describe protocols hosted on protocols.io. Read more information on sharing protocols at https://plos.org/protocols?utm_medium=editorial-email&utm_source=authorletters&utm_campaign=protocols.

As the corresponding author, your ORCID iD is verified in the submission system and will appear in the published article. PLOS supports the use of ORCID, and we encourage all coauthors to register for an ORCID iD and use it as well. Please encourage your coauthors to verify their ORCID iD within the submission system before final acceptance, as unverified ORCID iDs will not appear in the published article. *Only*  the individual author can complete the verification step; PLOS staff *cannot*  verify ORCID iDs on behalf of authors.

We look forward to receiving your revised manuscript.

Kind regards,

Rajiv R. Mohan, Ph.D.

Academic Editor

PLOS One

Journal requirements:

When submitting your revision, we need you to address these additional requirements.

1. Please ensure that your manuscript meets PLOS ONE's style requirements, including those for file naming. The PLOS ONE style templates can be found at

https://journals.plos.org/plosone/s/file?id=wjVg/PLOSOne_formatting_sample_main_body.pdf and

https://journals.plos.org/plosone/s/file?id=ba62/PLOSOne_formatting_sample_title_authors_affiliations.pdf

2. We note that you have indicated that there are restrictions to data sharing for this study. For studies involving human research participant data or other sensitive data, we encourage authors to share de-identified or anonymized data. However, when data cannot be publicly shared for ethical reasons, we allow authors to make their data sets available upon request. For information on unacceptable data access restrictions, please see http://journals.plos.org/plosone/s/data-availability#loc-unacceptable-data-access-restrictions.

Before we proceed with your manuscript, please address the following prompts:

a) If there are ethical or legal restrictions on sharing a de-identified data set, please explain them in detail (e.g., data contain potentially identifying or sensitive patient information, data are owned by a third-party organization, etc.) and who has imposed them (e.g., a Research Ethics Committee or Institutional Review Board, etc.). Please also provide contact information for a data access committee, ethics committee, or other institutional body to which data requests may be sent.

b) If there are no restrictions, please upload the minimal anonymized data set necessary to replicate your study findings to a stable, public repository and provide us with the relevant URLs, DOIs, or accession numbers. Please see http://www.bmj.com/content/340/bmj.c181.long for guidelines on how to de-identify and prepare clinical data for publication. For a list of recommended repositories, please see https://journals.plos.org/plosone/s/recommended-repositories. You also have the option of uploading the data as Supporting Information files, but we would recommend depositing data directly to a data repository if possible.

Please update your Data Availability statement in the submission form accordingly.

If the reviewer comments include a recommendation to cite specific previously published works, please review and evaluate these publications to determine whether they are relevant and should be cited. There is no requirement to cite these works unless the editor has indicated otherwise.

Additional Editor Comments:

Dear authors,

Your manuscript has completed peer-review process and comments are appended below. You will see that the reviewers though find research has merit but have expressed many serious concerns. Hence, I recommend adequate addressing of all concerns pointed by the reviewers through additional data and/or evidence from literature. Thank you

[Note: HTML markup is below. Please do not edit.]

Reviewers' comments:

Reviewer's Responses to Questions

**Comments to the Author**

1. Is the manuscript technically sound, and do the data support the conclusions?

The manuscript must describe a technically sound piece of scientific research with data that supports the conclusions. Experiments must have been conducted rigorously, with appropriate controls, replication, and sample sizes. The conclusions must be drawn appropriately based on the data presented.

Reviewer #1: Partly

Reviewer #2: Yes

**********

2. Has the statistical analysis been performed appropriately and rigorously?

Reviewer #1: Yes

Reviewer #2: Yes

**********

3. Have the authors made all data underlying the findings in their manuscript fully available?

The PLOS Data policy requires authors to make all data underlying the findings described in their manuscript fully available without restriction, with rare exception (please refer to the Data Availability Statement in the manuscript PDF file). The data should be provided as part of the manuscript or its supporting information, or deposited to a public repository. For example, in addition to summary statistics, the data points behind means, medians and variance measures should be available. If there are restrictions on publicly sharing data—e.g. participant privacy or use of data from a third party—those must be specified.

Reviewer #1: Yes

Reviewer #2: Yes

**********

4. Is the manuscript presented in an intelligible fashion and written in standard English?

PLOS ONE does not copyedit accepted manuscripts, so the language in submitted articles must be clear, correct, and unambiguous. Any typographical or grammatical errors should be corrected at revision, so please note any specific errors here.

Reviewer #1: Yes

Reviewer #2: Yes

**********

5. Review Comments to the Author

Please use the space provided to explain your answers to the questions above. You may also include additional comments for the author, including concerns about dual publication, research ethics, or publication ethics. (Please upload your review as an attachment if it exceeds 20,000 characters)

Reviewer #1: The authors present a retrospective analysis of factors associated with graft failure after DMEK. The following points would be a starting point to improve the manuscript:

1. Was superficial keratectomy carried out for any cases? Please report.

2. Graft variables are not sufficiently reported and could also influence graft survival. Please comment on donor age, storage duration, and endothelial cell density in greater detail.

3. Younger age is poorly defined in the study. Clarify the definition and quantify in the results. 70 vs. 75 is mentioned at line 321, but that age difference does not seem clinically significant.

4. While certainly a valid technique, suggesting DSAEK as an alternative in eyes with AL is not supported directly by the study. Would suggest to avoid specifically suggesting alternate technique without comparative data supporting this.

5. The timeline for graft failure would be useful to know (what % of grafts failed at 3 months vs. 6 months. vs. later). Suggestion to include survival plot.

Reviewer #2: Dear Authors,

thank you for this interesting and relevant manuscript. From my perspective, it would strengthen the study to include all patients—including those who underwent vitrectomy and those with suboptimal graft conditions—in the primary analysis to better reflect real-world clinical practice. A subsequent subgroup analysis excluding these cases could then provide additional insight into outcomes under more ideal conditions.

Such an approach would allow the reader to better appreciate both the “real-life” effectiveness and the results under optimized circumstances.

Kind regards!

**********

6. PLOS authors have the option to publish the peer review history of their article (what does this mean?). If published, this will include your full peer review and any attached files.

If you choose “no”, your identity will remain anonymous but your review may still be made public.

**Do you want your identity to be public for this peer review?**  For information about this choice, including consent withdrawal, please see our Privacy Policy.

Reviewer #1: No

Reviewer #2: No

**********

[NOTE: If reviewer comments were submitted as an attachment file, they will be attached to this email and accessible via the submission site. Please log into your account, locate the manuscript record, and check for the action link "View Attachments". If this link does not appear, there are no attachment files.]

To ensure your figures meet our technical requirements, please review our figure guidelines: https://journals.plos.org/plosone/s/figures

You may also use PLOS’s free figure tool, NAAS, to help you prepare publication quality figures: https://journals.plos.org/plosone/s/figures#loc-tools-for-figure-preparation.

NAAS will assess whether your figures meet our technical requirements by comparing each figure against our figure specifications.

# Author comment: Author response to Decision Letter 1

**DOI:** 10.1371/journal.pone.0352687.r002

29 May 2026

Response to Reviewers

Editor comments

1. Please ensure that your manuscript meets PLOS ONE's style requirements, including those for file naming. The PLOS ONE style templates can be found at

https://journals.plos.org/plosone/s/file?id=wjVg/PLOSOne_formatting_sample_main_body.pdf and

https://journals.plos.org/plosone/s/file?id=ba62/PLOSOne_formatting_sample_title_authors_affiliations.pdf

Reply: We ensured during revision that the paper conformed to these style requirements.

2. We note that you have indicated that there are restrictions to data sharing for this study. For studies involving human research participant data or other sensitive data, we encourage authors to share de-identified or anonymized data. However, when data cannot be publicly shared for ethical reasons, we allow authors to make their data sets available upon request. For information on unacceptable data access restrictions, please see http://journals.plos.org/plosone/s/data-availability#loc-unacceptable-data-access-restrictions.

Before we proceed with your manuscript, please address the following prompts:

a) If there are ethical or legal restrictions on sharing a de-identified data set, please explain them in detail (e.g., data contain potentially identifying or sensitive patient information, data are owned by a third-party organization, etc.) and who has imposed them (e.g., a Research Ethics Committee or Institutional Review Board, etc.). Please also provide contact information for a data access committee, ethics committee, or other institutional body to which data requests may be sent.

Please update your Data Availability statement in the submission form accordingly.

Reply: In France, the dataset cannot be shared on a repository, even when the patients have been anonymized. Legally, we cannot add to or change the original Data Availability statement, which is:

“The datasets generated during and/or analyzed during the current study are not publicly available according to French Law No. 2018-493 of June 20, 2018 on the protection of personal data (The General Data Protection Regulation (Regulation (EU) 2016/679) (GDPR: article 9) but are available from the Clinical Research Support Platform (Plateforme d’Appui à la Recherche Clinique [PARC]) of the Regional Central Hospital (CHR) of Metz-Thionville on reasonable request (email: projetrechercheclinique@chr-metz-thionville.fr, tel: +33 3 87 17 98 82). All nonarchived data is subject to daily backups while all archived data is subject to duplicate storage at two different sites. This data processing is compliant with a baseline reference methodology (MR001) to which the CHR MetzThionville signed a compliance commitment on October 8, 2018.”

Reviewer #1:

The authors present a retrospective analysis of factors associated with graft failure after DMEK. The following points would be a starting point to improve the manuscript:

Reply: Thank you very much for the time you have taken to review our manuscript, and for your helpful comments. We feel the manuscript is much improved after revision according to these and the other reviewer comments.

Comment 1:

Was superficial keratectomy carried out for any cases? Please report.

Reply: No, we did not perform any superficial keratectomies.

To address this point, we added the variable “Superficial keratectomy”and the fact that none were performed to Table 1, and the following text to the manuscript body:

Methods, Surgical techniques, Lines 189-191: “If the recipient eye had concomitant anterior corneal surface pathology, superficial keratectomy was conducted with alcohol delamination, followed by polishing with a cellulose sponge and irrigation.”

Comment 2:

Graft variables are not sufficiently reported and could also influence graft survival. Please comment on donor age, storage duration, and endothelial cell density in greater detail.

Reply: We have addressed this comment about each of these three variables as follows.

(i) Donor age: the donor age of our grafts was shown in Table 1, and Tables 3–4 indicate that indeed, younger donor age increases graft failure (Results, Lines 335, 349). This may reflect the greater handling involved with the scroll-prone grafts from younger donors and/or scrolling of the graft after preparation. These points are detailed in the Discussion (Line 512-526).

To address this comment, we expanded our description of the cohort:

Results, Preoperative and operative characteristics, Line 312: “Median graft donor age was 74 years.”

(ii) Graft-storage duration: we unfortunately found it difficult to retrieve these data for our study and were therefore not able to assess its role. However, existing studies on DMEK grafts that were stored in organ culture suggest that graft-storage duration is unlikely to markedly affect DMEK graft failure:

• Univariable analyses found that duration of DMEK-graft storage in organ culture does not significantly affect early DMEK graft failure20 or rebubbling20,16 rates.

• A multivariable analysis also showed that graft-storage time did not predict detachment rates.96

• While two multivariable analyses did show that 6-month96 and 5-year123 ECD after DMEK drops mildly by 0.7% and 1.2% per extra day in organ culture, respectively, another univariable analysis did not find this relationship 2 years after DMEK.17

20 Dunker et al. Br J Ophthalmol. 2021. doi:10.1136/bjophthalmol-2020-317041

16 Gerber-Hollbach et al. Cornea. 2017. doi:10.1097/ICO.0000000000001220

96 Rodríguez-Calvo De Mora et al. JAMA Ophthalmol. 2016. doi:10.1001/jamaophthalmol.2015.4499

123 Birbal et al. Cornea. 2020. doi:10.1097/ICO.0000000000002120

17 Peraza-Nieveset al. Cornea. 2017. doi:10.1097/ICO.0000000000001176

Thus, it seems that graft-storage duration has at best relatively mild effects on DMEK graft-success rates. To address this point, we added a new reference (Birbal et al., 2020, ref. 123):

Study Limitations, Line 578: “While we tested 20 covariates, we did not examine the contributions of other variables that may promote DMEK-graft failure, including surgeon learning curve [121], graft marking [122], graft storage conditions [96,123]...”

(iii) Endothelial cell density (ECD): all of our grafts had an ample preoperative ECD (all >2200 cells/mm2). Nonetheless, to address this point, we examined the effect of preoperative ECD on graft failure by conducting an additional univariable analysis. This showed that the median (interquartile range) preoperative ECD of the grafts that did and did not fail in the first postoperative year was 2460 (2380–2600) and 2550 (2400–2680) cells/mm2, respectively and the two groups did not differ significantly in terms of preoperative ECD (p=0.16).

To address this point, we added these ECD data to our analyses. This changed the number of pre/perioperative independent variables we studied from 19 to 20 (see Lines 43, 122, 259, 579). Tables 1 and 2 now include the preoperative ECD data. We also added the following texts to the revised manuscript:

Methods, Preoperative variables, Lines 161-163: “Preoperative graft ECD was determined by manual counting under an optic microscope by an eye-bank technician followed by a recount by another technician.”

Methods, Surgical techniques, Lines 175-180: “These preoperative ECD measurements were made just before transport, which occurred 3–4 weeks after button harvest. For transport, the buttons were placed in dextran-containing transport medium (CorneaJet; Eurobio) and shipped to our hospital the day before surgery. They were exposed to the transport medium for 3 days. DMEK grafts were generated from the corneoscleral buttons immediately before surgery.”

Comment 3:

Younger age is poorly defined in the study. Clarify the definition and quantify in the results. 70 vs. 75 is mentioned at line 321, but that age difference does not seem clinically significant.

Reply: We agree and have reduced and revised the Abstract and Discussion to reflect this. The following texts were deleted from the Abstract and Discussion:

Abstract: “Younger donor age weakly predicted DMEK-graft failure, possibly due to tighter scrolling of younger grafts.”

Discussion, relationship with donor age: “Nonetheless, our finding suggests that as long as they have ample endothelial-cell reserve, older donors may be preferrable to younger donors for DMEK, particularly if the surgeon is still in training [9].”

The remaining Discussion text was modulated:

Discussion, Relationship with donor age, Lines 522-535: “It should be noted, however, that graft-unscrolling/positioning difficulties did not associate with graft-donor age in our univariable analyses (Table 4). An alternative possibility is that graft scrolling inflicts detrimental ECL-inducing mechanical forces on the endothelial cells before transplantation (Fig. 3, straight top right red arrow). For example, the tight scrolling of young grafts (with endothelium on the outside [98]) could compress and rub the cells on the inside layers. These mechanical forces may both damage the cells physically and initiate detrimental mechanosignalling in the endothelial cells that induces their death [99].

However, it should be emphasized that the effect of donor age in our study was mild (OR=0.93) and the median ages of the patients who did and did not undergo graft failure were 70 and 75 years, respectively. Moreover, many other studies have not observed a link between graft-donor age and graft failure itself (S1 Table) [20,28,30,31,36,37] or its surrogates, namely, rebubbling, graft detachment [20,30,37,42,100–105], and ECL [10,11,18,54,96,100]. Thus, the clinical relevance of this finding may be limited.”

Comment 4:

While certainly a valid technique, suggesting DSAEK as an alternative in eyes with AL is not supported directly by the study. Would suggest to avoid specifically suggesting alternate technique without comparative data supporting this.

Reply: We believe that identifying this problem of long AXL requires us to suggest alternative strategies to improve outcomes. We are currently conducting DSAEK in eyes with AXL≥25 that are otherwise eligible for DMEK, and will publish the outcomes of these cases in due course. However, we agree that we should reduce the emphasis on DSAEK as an alternative to DMEK in AXL≥25 mm cases.

To address this comment, we removed the following text from the Abstract:

“DSAEK may be more appropriate for such eyes.”

Comment 5:

The timeline for graft failure would be useful to know (what % of grafts failed at 3 months vs. 6 months. vs. later). Suggestion to include survival plot.

Reply: Thank you for this comment. To address it, we added the following texts to the Statistics and Results sections and created a new Fig 3 to show the graft survival curve (the original Fig 3 is now designated Fig 4). We also added/clarified details regarding follow-up of the cohort:

Abstract, Lines 40-42: “This retrospective cohort study included consecutive eyes that underwent DMEK in 2015-2023 in a regional referral hospital and were followed for at least 12 months. DMEK-graft failure was defined as need for regrafting during follow-up.”

Abstract, Lines 49-50: “Median (range) follow-up was 24 (12–29) months, during which 15 grafts (9%) failed.”

Methods, Surgical techniques, Lines 235–237: “If non-adhesion continued despite up to four rebubblings, corneal edema persisted after 3 months, and/or new corneal edema arose during follow-up, the graft was considered to have failed”

Methods, Collected variables, Lines 250-251: “Graft failure was defined as need for a new graft during follow-up due to any reason”

Statistics, Lines 273-274: “To depict the time course of graft failures, Kaplan-Meier survival analysis was conducted.”

Results, Lines 302–303: “The median (IQR; range) follow-up duration of these 170 eyes was 24 (12–24; 12–29) months.”

Results, Postoperative outcomes, Lines 322-326: “During follow-up, 15 (9%) of the eyes had experienced graft failure (Table 1). Of these 15 grafts, seven (47%), three (20%), two (13%), and three (20%) failed at ≤3 months, 4–6 months, 7–12 months, and >12 months, respectively. The median (IQR) duration to graft failure was 5 (2–9) months. Fig 3 shows the survival curve of all grafts.”

Figure legends, Lines 1034-1037:

“Fig 3. DMEK graft survival curve.

Fig 4. Schematic depiction of potential mechanisms by which pre/perioperative factors could induce DMEK graft failure.”

Reviewer #2:

Dear Authors,

thank you for this interesting and relevant manuscript. From my perspective, it would strengthen the study to include all patients—including those who underwent vitrectomy and those with suboptimal graft conditions—in the primary analysis to better reflect real-world clinical practice. A subsequent subgroup analysis excluding these cases could then provide additional insight into outcomes under more ideal conditions.

Such an approach would allow the reader to better appreciate both the “real-life” effectiveness and the results under optimized circumstances.

Kind regards!

Reply: Thank you very much for your time and helpful suggestion.

To address this comment, we repeated the multivariable analysis with all 188 eyes, including the 17 eyes with graft-dissection problems, the single eye with vitrectomy, and the four eyes with missing AXL and/or ACD values. Almost exactly the same results were obtained.

To address this, we added the following texts and new S3 Table (the original S3 and S4 Tables are now designated S4 and S5 Tables, respectively, see revised Supplementary Data):

Methods, Patient selection, Lines 152-153: “To assess the effect of these exclusions on our findings, we repeated our multivariable analysis with all consecutive patients.”

Statistics, Lines 274-277: “To assess the effect of excluding cases with graft-dissection problems, vitrectomy, missing data, and complicated rejection/infection features on our multivariable-analysis findings, we repeated this analysis with all consecutive patients.”

Results, Multivariable analyses, Lines 359-361: “To assess the effect of excluding the 18 eyes with graft-dissection problems, vitrectomy, missing data, and unusual rejection/infection features from our study cohort, we repeated the multivariable analysis. Very similar results were obtained (S3 Table).”

Attachment Submitted filename: Response to Reviewers.docx

# Peer review: Decision Letter 1

**DOI:** 10.1371/journal.pone.0352687.r003

**Authors:** Rajiv R. Mohan

15 Jun 2026

Preoperative and perioperative factors that predict graft failure 1 year after Descemet membrane endothelial keratoplasty

PONE-D-26-11203R1

Dear Dr. %PeroneE%,

We’re pleased to inform you that your manuscript has been judged scientifically suitable for publication and will be formally accepted for publication once it meets all outstanding technical requirements.

Within one week, you’ll receive an e-mail detailing the required amendments. When these have been addressed, you’ll receive a formal acceptance letter and your manuscript will be scheduled for publication.

An invoice will be generated when your article is formally accepted. Please note, if your institution has a publishing partnership with PLOS and your article meets the relevant criteria, all or part of your publication costs will be covered. Please make sure your user information is up-to-date by logging into Editorial Manager at Editorial Manager® and clicking the ‘Update My Information' link at the top of the page. For questions related to billing, please contact billing support.

If your institution or institutions have a press office, please notify them about your upcoming paper to help maximize its impact. If they’ll be preparing press materials, please inform our press team as soon as possible -- no later than 48 hours after receiving the formal acceptance. Your manuscript will remain under strict press embargo until 2 pm Eastern Time on the date of publication. For more information, please contact onepress@plos.org.

Kind regards,

Rajiv R. Mohan, Ph.D.

Academic Editor

PLOS One

Additional Editor Comments (optional):

Dear authors,

I am happy to inform that your revisions/changes adequately addressed all concerns. The manuscript is accepted for publication. Congratulations!

Reviewers' comments:

Reviewer's Responses to Questions

**Comments to the Author**

1. If the authors have adequately addressed your comments raised in a previous round of review and you feel that this manuscript is now acceptable for publication, you may indicate that here to bypass the “Comments to the Author” section, enter your conflict of interest statement in the “Confidential to Editor” section, and submit your "Accept" recommendation.

Reviewer #2: All comments have been addressed

**********

2. Is the manuscript technically sound, and do the data support the conclusions?

The manuscript must describe a technically sound piece of scientific research with data that supports the conclusions. Experiments must have been conducted rigorously, with appropriate controls, replication, and sample sizes. The conclusions must be drawn appropriately based on the data presented.

Reviewer #2: Yes

**********

3. Has the statistical analysis been performed appropriately and rigorously?

Reviewer #2: Yes

**********

4. Have the authors made all data underlying the findings in their manuscript fully available?

The PLOS Data policy requires authors to make all data underlying the findings described in their manuscript fully available without restriction, with rare exception (please refer to the Data Availability Statement in the manuscript PDF file). The data should be provided as part of the manuscript or its supporting information, or deposited to a public repository. For example, in addition to summary statistics, the data points behind means, medians and variance measures should be available. If there are restrictions on publicly sharing data—e.g. participant privacy or use of data from a third party—those must be specified.

Reviewer #2: Yes

**********

5. Is the manuscript presented in an intelligible fashion and written in standard English?

PLOS ONE does not copyedit accepted manuscripts, so the language in submitted articles must be clear, correct, and unambiguous. Any typographical or grammatical errors should be corrected at revision, so please note any specific errors here.

Reviewer #2: Yes

**********

6. Review Comments to the Author

Please use the space provided to explain your answers to the questions above. You may also include additional comments for the author, including concerns about dual publication, research ethics, or publication ethics. (Please upload your review as an attachment if it exceeds 20,000 characters)

Reviewer #2: Dear Authors,

thank you for adressing all comments.

Kind regards!

**********

7. PLOS authors have the option to publish the peer review history of their article (what does this mean?). If published, this will include your full peer review and any attached files.

If you choose “no”, your identity will remain anonymous but your review may still be made public.

**Do you want your identity to be public for this peer review?**  For information about this choice, including consent withdrawal, please see our Privacy Policy.

Reviewer #2: No

**********

# Editor report: Acceptance letter

**DOI:** 10.1371/journal.pone.0352687.r004

**Authors:** Rajiv R. Mohan

PONE-D-26-11203R1

PLOS One

Dear Dr. Perone,

I'm pleased to inform you that your manuscript has been deemed suitable for publication in PLOS One. Congratulations! Your manuscript is now being handed over to our production team.

At this stage, our production department will prepare your paper for publication. This includes ensuring the following:

* All references, tables, and figures are properly cited

* All relevant supporting information is included in the manuscript submission,

* There are no issues that prevent the paper from being properly typeset

You will receive further instructions from the production team, including instructions on how to review your proof when it is ready. Please keep in mind that we are working through a large volume of accepted articles, so please give us a few days to review your paper and let you know the next and final steps.

Lastly, if your institution or institutions have a press office, please let them know about your upcoming paper now to help maximize its impact. If they'll be preparing press materials, please inform our press team within the next 48 hours. Your manuscript will remain under strict press embargo until 2 pm Eastern Time on the date of publication. For more information, please contact onepress@plos.org.

You will receive an invoice from PLOS for your publication fee after your manuscript has reached the completed accept phase. If you receive an email requesting payment before acceptance or for any other service, this may be a phishing scheme. Learn how to identify phishing emails and protect your accounts at https://explore.plos.org/phishing.

If we can help with anything else, please email us at customercare@plos.org.

Thank you for submitting your work to PLOS ONE and supporting open access.

Kind regards,

PLOS ONE Editorial Office Staff

on behalf of

Dr. Rajiv R. Mohan

Academic Editor

PLOS One
